# Supplementary material for: A Sizer model for cell differentiation in Arabidopsis thaliana root growth
Source: Mol Syst Biol. 2018 Jan 11;14(1):e7687. doi: 10.15252/msb.20177687 (PMC5787709; doi:10.15252/msb.20177687)
Supplement: Supplementary file 1 — Appendix [file MSB-14-e7687-s001.pdf]

Appendix. Supporting Information for Manuscript:

A Sizer model for cell differentiation in *Arabidopsis thaliana* root growth

Authors: Irina Pavelescu[a,b], Josep Vilarrasa-Blasi[a,&], Ainoa Planas-Riverola[a], Mary-Paz González-García[a,#] and Ana I. Caño-Delgado[a,1,2], Marta Ibañes[b,c,1,2].

Affiliations:

[a]Department of Molecular Genetics, Center for Research in Agricultural Genomics (CRAG) CSIC-IRTA-UAB-UB, Campus UAB, Bellaterra (Cerdanyola del Vallès), E-08193 Barcelona, Spain. [b]Department of Física de la Matèria Condensada, Physics, University of Barcelona, 08028 Barcelona, Spain, [c]Universitat de Barcelona Institute of Complex Systems (UBICS), Barcelona, Spain

& Current address: Carnegie Institution for Science, Department of Plant Biology, 260 Panama street, Stanford, California 94305, USA

# Current address: Centro Nacional de Biotecnología-CSIC, Cantoblanco, E-28049 Madrid, Spain

Contact: [1]Equal contribution. [2]To whom correspondence should be addressed:

Email: mibanes@ub.edu, ana.cano@cragenomica.es

(A.I.C.-D.). Tel: +34 93 563 66 00 Ext. 3210; Fax: +34 93 563 66 01

(M.I). Tel. +34 93 403 91 77

Contents

S0. Introduction

S1. Supporting Methods

S1.A.Theoretical framework for stationary root growth

S1.B. Automated fitting of individual wild type plants

S1.C.Validation of the automated fitting on individual roots

S1.D. Computational validation of the methodology used for extraction of dynamical parameters

S1. E. Theoretical predictions of the Ruler, Timer and Sizer models

S1. F. Computations used in Fig. 4

S2. Supplementary Notes

S2.A. On the results concerning the method to set the boundary between the MZ and EZ

S2.B. On the agreement of extracted average quantitative values of Arabidopsis roots with previously reported data

S3. Program codes

S3. A. Code for the automated fitting of individual roots

S0. Introduction

The pleiotropic defects exhibited by known mutants and hormone-treated roots can be explained at a qualitative level by different mechanisms on how cells decide to stop elongating. The short root phenotype exhibited by mutant plants insensitive to Brassinosteroid signalling can be used as an example. These short roots exhibit a reduced meristematic activity and short mature epidermal and cortical cell lengths (González-García *et al*, 2011a). Hence, Brassinosteroid signalling impinges in the two factors dictating root growth. But how do the shorter mature cell lengths arise in these plants? The average cell elongation rate in these roots is decreased but the average time cells spend elongating is not altered compared to the wild type (Cole *et al*, 2014). In addition, the length of the meristem and elongation zones is reduced also (González-García *et al*, 2011b; Hacham *et al*, 2011).

The short mature cell length phenotype can be explained in very different ways using all these data. At least three alternative explanations are possible. First, mature cells are shorter because cells elongate more slowly but during the same average time (Beemster & Baskin, 1998). According to this explanation (Timer mechanism), brassinosteroid signalling does not control what triggers cells to stop elongating (time), but it controls root growth by modifying meristematic cell division and cell elongation processes only, as recently suggested (Kang *et al*, 2017). A second possible explanation is that mature cells in these plants are shorter because they elongate more slowly until reaching a shorter threshold position (distance from QC, or from MZ). According to this description (Ruler mechanism), brassinosteroid signalling controls the threshold for differentiation (the cell position), besides the cell elongation rate and the meristematic activity. In addition, we may suggest that there may be a mechanism that ensures the robustness of the average time cells spend elongating. Interestingly, this average time is robust to many different hormone treatments (Beemster & Baskin, 1998). For instance, treatment with phytohorme auxin drives unaltered average time elongating, but altered root growth, cell elongation rates and reduced MZ and EZ lengths (Mähönen *et al*, 2014). A third different alternative description can be also envisaged. In this third description cells stop elongating when reaching a threshold length (Sizer mechanism), independently of how fast is the cell elongation rate or the meristematic activity. In this third scenario, brassinosteroid signalling controls the threshold length, and also the cell elongation rate and the meristematic activity. The average time cells spend elongating is not altered because cells elongate at a shorter rate but until a smaller final length. Also in this scenario, an additional mechanism ensuring the robustness of the average time of elongation may be claimed.

Therefore, solely from the qualitative data indicated, it becomes impossible to discard any of the three mechanisms and pinpoint only one as the most plausible one. The same type of explanations can be expected to be applied to the pleiotropic phenotypes exhibited by roots with other signalling pathways being altered.

S1. Supporting Methods.

S1.A. Theoretical framework for stationary root growth.

Herein we show that knowledge of the meristematic activity, and cell elongation dynamics is not sufficient to know how the root grows. Knowledge of how cells decide to stop elongating is also required.

Root growth is a very dynamical process, with cells constantly moving through each zone. After being produced in the meristem and divided, cells transit the elongation zone, increasing their length, and finally differentiate. When root growth reaches the steady state, meristematic and elongation zones achieve their maximal number of cells and length. From this moment, only the mature zone keeps increasing, contributing to root growth. We simplified further the dynamics of cells in the stationary root growth regime in two consecutive steps. First cells divide. After a while, cells stop dividing and start elongating until differentiating. We assumed that a stationary average production rate of cells at the meristem per cell file could be defined, which we termed R_{prod}. A rather constant average production rate of cells is supported by previous measurements in Arabidopsis thaliana primary root (Beemster & Baskin, 1998; Ivanov & Dubrovsky, 1997). In this theoretical framework we assumed that cells leave the meristem and enter the elongation zone in periodic time intervals given by $1/R_{prod}$. However, the computational models presented in the main text do not have this simplification and instead take into account variable time intervals between successive cells entering the EZ. We also assumed that cells elongate with a constant elongation rate, r_{elong}, such that the length l of any cell in the elongation zone follows the dynamics $dl/dt = r_{elong}l$, which results in

$$l(t) = l_0 e^{r_{elong}(t-t_i)} \qquad (1)$$

where l_0 is the size of the cell when it exists the meristem and enters the elongation zone at time t_i. The approximation of a constant cell elongation rate is consistent with previous measurements of the cell lengths in the elongation zone (Band *et al*, 2012; Cole *et al*, 2014). Nevertheless, this approximation is not relevant for the Ruler and Sizer models that will be used.

According to Eq. 1, the length of mature cells is

$$l_{diff} = l_0 e^{r_{elong}T} \qquad (2)$$

where T is the time during which the cell has elongated (i.e. the time spent in the elongation zone). At the stationary regime, the sizes of the meristem (L_{MZ}) and the elongation zone (L_{EZ}) are constant. Accordingly, the length of the root increases linearly with time as

$$L(t) = l_{diff} R_{prod} t + L_{EZ} + L_{MZ}. \qquad (3)$$

In agreement, wild type roots exhibit a linear root growth with time from day 4 post germination (Fig. 1). According to Eq. 3, the root growth rate ($R_{growth} \equiv dL(t)/dt$) is given by

$$R_{growth} = R_{prod} l_{diff}. \tag{4}$$

According to Eq. 1, the lengths of cells at any given time t along a cell file correspond to $l_i(t) = l_0 e^{r_{elong}(t-t_i)}$ where $i = 1,2,\ldots,N_{EZ}$ denotes the cell number position from the meristem, t_i is the time at which cell i entered into the elongation zone and N_{EZ} is the number of cells in the elongation zone. Since our framework assumes a constant rate R_{prod} of entrance of cells at the elongation zone, then $t_{i+1} = t_i - 1/R_{prod}$. Introducing this time iteratively in the expression of $l_i(t)$ results in $l_i(t) = \widetilde{l_0(t)} e^{r_{elong} i/R_{prod}}$ where $\widetilde{l_0(t)} = l_0 e^{r_{elong}(t-t_1)}$ and $t_1 \in [t - 1/R_{prod}, t]$. Hence, the cell length profile along a cell file follows an exponential profile as a function of the cell position i (Cole *et al*, 2014). Cole et al. used the representation with cortex cell number position and found that Col-0 WT plants and 5 mutants hold such an exponential profile (Cole *et al*, 2014). They indicated that such behaviour was consistent with an exponential lengthening of cells and a constant meristematic activity, as we consider. We found such exponential relationship (linear in logarithmic scale) for the lengths of epidermal and cortex cells in wild type roots in all days analyzed (Fig. 1, Appendix Figs. S2 and S3). This relationship can be simplified by assuming $t_1 \approx t$, which drives

$$l_i = l_0 e^{r_{elong} i/R_{prod}} \equiv l_0 r_{EZ}^i \tag{5}$$

and where the elongation factor $r_{EZ} \equiv \frac{l_{i+1}}{l_i} = e^{\frac{r_{elong}}{R_{prod}}}$ that measures how much longer is the adjacent cell with respect to the previous one, has been defined. Hence, from the slope of the length profiles in logarithmic scale of wild type roots we can extract the value of r_{EZ} (see Automated Quantification of wild type roots). Appendix Figs. S1 and S2 show that r_{EZ} in wild type roots in the stationary regime remains rather constant along time.

The framework enables to set a relationship for the length of the elongation zone as well:

$$L_{EZ} = \sum_{i=0}^{N_{EZ}-1} l_i = l_0 \frac{1-r_{EZ}^{N_{EZ}}}{1-r_{EZ}} = l_0 \frac{1-e^{r_{elong} N_{EZ}/R_{prod}}}{1-e^{r_{elong}/R_{prod}}} \tag{6}$$

where Eq. 5 and $r_{EZ} \equiv \frac{l_{i+1}}{l_i} = e^{\frac{r_{elong}}{R_{prod}}}$ have been used.

Hence, in order to predict the length of mature cells (Eq. 2) and thereby root growth (Eq. 4), we need to know the cell dynamics and features l_0, r_{elong}, R_{prod} (or in terms of l_0, r_{EZ}, r_{elong}) and also T. Instead of T, we could use N_{EZ} (since according to our framework T and N_{EZ} are related by $T = \frac{N_{EZ}}{R_{prod}}$) or L_{EZ} (see Eq. 6). Hence, to determine root growth l_0, r_{elong}, R_{prod} data are not sufficient and additional information is required. Setting a differentiation rule on how cells decide to stop elongating enables to know this additional information (see section S1.E).

.

S1.B. Automated fitting of individual wild type plants we reasoned that the cell elongation factor r_{EZ} in an individual root file could be extracted from the slope of linear fittings of the logarithm of the cell length as a function of the cell position. Hence this factor indicates how much longer is on average a cell with respect to its adjacent one. Yet to perform such fitting it was necessary to first decide where the elongation zone started. We decided to develop a new method to extract this position, which was based on the two trend behavior we observed on cell lengths. We reasoned that the cell where the second trend starts could be used as a robust measurement of where the elongation zone initiates. Therefore, we related the first trend with the dividing meristem, and the second trend with the elongation zone (Appendix Fig. S3A). An automated iterative procedure to extract this boundary position was established as detailed here below. We developed a code (see code in S3.A) run in Octave (version 4.2.1) (Eaton *et al*) to automatically find the intersection point between the two zones.
For the cell length data measurements extracted for each analyzed root j, we applied a linear fit 'p_j(z)' to the logarithms of the length of cells from position 1 to z closest to the QC and another linear fit 'v_j(z)' to the logarithms of the length of cells from position z+1 to N. N was given by the position previous to the first differentiated cell, which was dictated by the appearance of the hair bulge. From these two fittings, first estimations of the cell elongation factors in the meristematic ($r_{MZ,j}$) and elongation ($r_{EZ,j}$) zones, the length of the first cell in the meristem ($l_{0MZ,j}$) and the length of the last cell in the meristem ($l_{0EZ,j}$) were extracted as (Appendix Fig. S3A):

$$r_{MZ,j} = \mathrm{e}^{\text{slope of } \mathrm{p}_j(z)} \quad (7)$$

$$r_{EZ,j} = \mathrm{e}^{\text{slope of } \mathrm{v}_j(z)} \tag{8}$$

$$l_{0MZ,j} = \mathrm{e}^{\text{intercept of } \mathrm{p}_j(z)} \tag{9}$$

$$l_{0EZ,j} = \mathrm{e}^{\text{intercept of } \mathrm{v}_j(z)} \tag{10}$$

where the subindex j emphasizes that the fittings and extraction of parameter values was done for each individual root.

It is worth to stress that these estimations depend on the value of z; yet this dependence has been not explicitly indicated on each left-hand side term to simplify the notation. The v_j fitting was done by shifting the ordinal values of the cell position to 1 up to N-z, and hence $l_{0EZ,j}$ was directly given by its intercept (Appendix Fig. S3A).

These two linear fittings (and hence the estimation of parameters according to Eqs. 7-10) for each individual root were done iteratively for each scalar value of z from 5 to N-3 (we considered 5 as the minimum number of cells in the meristem and 3 as the minimum number of cells in the elongation zone). For each individual root, the pair of fittings extracted at each z value was checked to evaluate whether the following conditions were fulfilled simultaneously:

- the length of the cell in the position z-2 in the meristem is larger than the length of the cell in position 0 in the elongation zone:

$$\frac{l_{0MZ} {r_{MZ}}^{z-2}}{l_{0EZ} r_{EZ}} > 1 \tag{11}$$

- the length of the cell in the position z-1 in the meristem is shorter or equal to the length of the cell in position 0 in the elongation zone:

$$\frac{l_{0MZ} {r_{MZ}}^{z-1}}{l_{0EZ} r_{EZ}} \leq 1 \tag{12}$$

- the length of the cell in the position z in the meristem is shorter than the length of the cell in position 0 in the elongation zone:

$$\frac{l_{0MZ} {r_{MZ}}^{z}}{l_{0EZ} r_{EZ}} < 1 \tag{13}$$

- the number of cells in the meristem has to be equal or higher to the number of cells in the elongation zone: $N_{MZ,j} \geq N_{EZ,j}$. This is based on previous measurements that indicate that the elongation zone is smaller (González-García *et al*, 2011a; Mähönen *et al*, 2014).

The first z value for which all these conditions were fulfilled was the one selected as the intersection point between the meristematic and elongation zones (hereafter named z_0), such that the number of cells in the meristematic zone was set to be given by

$$N_{MZ,j} = z_0 \quad (14)$$

and the number of cells in the elongation zone was set as

$$N_{EZ,j} = N - z_0. \quad (15)$$

In some cases (5% approximately of roots) it was impossible to find a z value for which all the above rules were fulfilled and so another criterion, with less constraints, was applied, to select for a z_0 value in these roots. This criterion was identical with the previous described one, except for the intersection conditions, which omit Eq. 13. In this way we allowed the fits to intersect in the interval (z-2, z), without restraining it to (z-2, z-1) interval.

Even so, there were still 2% of the plant roots that could not be analyzed using these criteria (i.e. for no z value the criteria were fulfilled). To solve this problem, we developed a third criterion based on maximizing the function $F(z)$ defined as

$$F(z) = \sqrt{\left|{R_p}^2 + {R_v}^2\right|} \quad (16)$$

where ${R_p}^2$ and ${R_v}^2$ are the coefficients of determination of each fit. The coeficient of determination R^2 is defined as 1 minus the ratio between the residual sum of squares and the total sum of squares: $R^2 \equiv 1 - \frac{\sum_i (y_i - f_i)^2}{\sum_i (y_i - \bar{y})^2}$, where y_i stand for experimental data, f_i for the corresponding fitted (predicted) values according to the least square method and $\bar{y}$ for the mean of the experimental data (computed in Octave). The selected value of z (z_0) was the one maximizing $F(z)$. In this way, even if one fit was not the best solution for the given points, the combination of the two fits was the best solution for the whole system of points. In this way we chose only the value that gives the best general solution.

Once a z value was selected (z_0) according to the above criteria, we set the values of $N_{MZ,j}$ and $N_{EZ,j}$ according to Eqs.14,15, respectively. Final values for $r_{MZ,j}$, $r_{EZ,j}$, $l_{0MZ,j}$ and $l_{0EZ,j}$ for each individual plant were calculated as described in Eqs. 7-10 for the fittings corresponding to $z = z_0$.

After calculating the values for each parameter for each plant, we observed that in some cases the difference $\Delta r = r_{EZ,j} - r_{MZ,j}$ was very small (smaller than 0.08), so we considered that there was no difference between the first and the second zones and that all cells belonged to the meristematic zone, setting $N_{MZ,j} = N$ with elongation factor $r_{MZ,j}$ and length of first meristematic cell $l_{0MZ,j}$ given by a new linear fitting applied

to all data, from 1 to N. This situation was more frequent (25%) in the roots from first day post germination.

Parameter values for each individual plant were exported into Excel files. The measured length of the last elongated cell (l_{diff}) was also automatically extracted from each plant individually, as an appendix to the main code and exported into Excel files. Post measurements analysis were done in Excel (Microsoft Office), R (R Core Team (2016). R: A language and environment for statistical computing.), MATLAB R2009b (The MathWorks Inc., Natick, MA, 2000) and SigmaPlot 11.0 (Systat Software).

S1.C. Validation of the automated fitting on individual roots.

As a first validation, we evaluated the distributions of determination coefficients R^2 for the linear regressions applied to MZ and EZ (Appendix Fig. S3B). In the elongation zone, the median value was above 0.9, indicating a good linear approximation to the data. The only exception was the distribution of day 1, which was wider than the rest and with a lower median value (0.85). This was due to a higher variability in cell lengths at this time point. In the meristematic zone, cell length was maintained almost constant, with a slope close to 0, which generated low values for the determination coefficient.

Second, we verified that the extracted values from the linear regressions applied to the meristematic and elongation zones were consistent to the real, directly measurable, values of the individual plant root files. First, for each individual root we compared direct measurements of the length of the first meristematic cell with the extracted value $l_{0MZ,j}$ (the intercept of the selected fitting performed in the meristematic zone, Eq. 9 for $z=z_0=N_{MZ,j}$) (Appendix Fig. S3C). The distribution of values showed no statistical significant differences, although the distribution for the extracted values was narrower. This is to be expected since the intersection point of the linear regression with the vertical axis is dictated by the length and position of all the cells in the meristem, reducing its variability. Comparison of the two values for each individual root lied roughly in the bisectrix (Appendix Fig. S3C bottom). Next, we compared the measured value of the length of the last meristematic cell (corresponding to the N_{MZ} position from QC) to the value extracted from the linear fitting of the elongation zone (taken as the intercept value, Eq. 10 for $z=z_0=N_{MZ,j}$) for each individual root file (Appendix Fig. S3D). No statistical significant difference between the two distributions was found and

the pair of values for each root lied around the bisectrix. Also, a good agreement was found (Appendix Fig. S3E) when comparing the measured value of the length of the last meristematic cell with the value predicted from the linear fitting of the meristematic zone as

$l_{0EZ\,\text{predicted,j}} = l_{0MZ,j}\, r_{MZ,j}{}^{N_{MZ,j}-1}$

where the values of the meristematic fitting were used (Eqs. 7 and 9 for $z=z_0=N_{MZ,j}$) as well as the boundary value $N_{MZ,j}$ given by the two fittings for each individual plant. Finally, we compared the measured length of mature cells with the value predicted from the fitting of the elongation zone as

$l_{diff\,\text{predicted,j}} = l_{0EZ,j}\, r_{EZ,j}{}^{N_{EZ,j}}$

using the values extracted from the fittings (Eqs. 8 and 10 for $z=z_0=N_{MZ,j}$, and $N_{EZ,j}$) extracted for each individual root. Again, data were in good agreement (Appendix Fig. S3F). Taken together, these data support that our procedure gives consistent results.

To support that cells in the first regime can be used as a good measurement of the dividing meristem, we counted the number of epidermal cells corresponding to the YFP expression domain of RP5Sa promoter and the number of epidermal cells of the first trend at different days in pRP5Sa:BRI1:YFP; *bri1* plants (Fig. 5C, inset). Our results show that both of them are in very good agreement (Table EV6).

S1.D. Computational validation of the methodology used for extraction of dynamical parameters

Since the computational models provide a good first approximation for root file growth and exhibit heterogeneity (i.e. variability among cells and root files), we used them to evaluate whether the procedures detailed in previous sections S1A and S1B to infer dynamical information (such as cell elongation rate and time spend in the EZ, from morphological data on the cell length profile along a root file and from the length of the root over time) provide reliable results in the heterogeneous context of the computational data.

The evaluation was performed by first applying the methods to the data coming from the simulated root files and then comparing the resulting inferred dynamical information to the exact one of the computational roots, which was directly computed in the simulations. We performed the validation for all three models (Appendix Figs. S5-S7).

Because the computational models do not simulate the dynamics in the MZ, the N_{EZ} value was measured directly in the simulations from how many cells were still elongating. Thus, the way N_{EZ} was measured in the simulated roots was distinct from that explained in S1B.

To extract the R_{prod} output value, we performed both the Approach 1 (Appendix Figs. S5-S7B) and Approach 2 (Appendix Figs. S5-S7A), according to the equations in row K in Table EV2. r_{elong} and t_{resid} values were computed according to rows M and O in Table EV2. Using Approach 2, a distribution of values could be obtained for all three parameters, in contrast to Approach 1, when a unique value could be extracted for R_{prod}. If our methodology to infer dynamical information is useful when there is variability between cells, then the exact (named input in Appendix Figures S5-S7 for simplicity) and inferred (named output in Appendix Figs. S5-S7) values should be similar. Indeed, this was observed when we compared input and output data for Approach 1 (Appendix Figs. S5-S7B) and Approach 2 (Appendix Figs. S5-S7A).

Moreover, we found a good correlation between input and output values of each simulated root file (Appendix Figs. S5-S7A, left panel). These results support that the methods presented in S1A and S1B for extracting dynamical information on root growth dynamics can drive reliable results when applied on our computational models, which contain variability between cells and root files. This, together with our computational models being a good first approximation to wild type root growth, suggests these methods can be expected to drive also reliable information when applied on real roots.

In summary, for all models (Ruler, Timer and Sizer), the data presented in Appendix Fig. S5-7 was computed as shown below:

Inputs values (Appendix Fig. S5-7A)

$R_{prod\_input\_j} = \langle R_{prodji} \rangle$, the average of all cellular $R_{prod\ i}$ values in a plant j

$r_{elong\_input\_j} = \langle r_{elongji} \rangle$, the average of all cellular $r_{elong\ i}$ values in a plant j

$t_{resid\_input\_j} = \langle t_{resid\_computed\_ij} \rangle$, the average of all cellular t_{resid_i} values in plant j, where $t_{resid\_computed\_ij} = t_{diff\_ij} - t_{born\_ij}$.

Output values (Appendix Fig. S5-7A)

Approach 2:

$R_{prod\_output\_j}=R_{growth\_j}/l_{diff\_j}$,with R_{growth_j} extracted as the slope of the linear regression applied to the sum of newly incorporated mature cells versus the incorporation time (as in Fig. 1C). The slope is based on 200 cells, on average.

l_{diff_j}=last temporally mature cell; first cell in the mature zone counting from the EZ.

r_{EZ_j} was extracted from the slope of the linear egression applied to the cell lengths (l_i) in the elongation zone versus their position from the end of the meristematic zone (N_{EZ}).

N_{EZ_j} was calculated as the number of cells in the elongation zone a the end of the simulation time in plant j.

$r_{elong\_output\_j}=\ln r_{EZ\_output\_j}*R_{prod\_output\_j}$

$t_{resid\_output\_j}=N_{EZ\_j}*\ln r_{EZ\_j}/r_{elong\_output\_j}$

Output values (Appendix Fig. S5-7B)

Approach 1

$R_{prod\_output\_j\_1}=R_{growth\_j\_1}/<l_{diff\_j},>$, with $R_{growth\_j\_1}$ extracted as $<slope_j>$, where $slope_j$ is the slope of the linear regression applied to the sum of newly incorporated mature cells versus the incorporation time, in plant j (as in Fig. 1C). The slope is based on 200 cells, on average.

$R_{prod\_output\_j\_2}=R_{growth\_j\_2}/<l_{diff\_j},>$, with $R_{growth\_j\_2}$ extracted as the slope of the linear regression applied to the <root_length_j> at 4 simulated timepoints (1,2, 3 and 4).

$r_{elong\_output\_j\_1}=\ln r_{EZ\_output\_j}*R_{prod\_output\_j\_1}$

$t_{resid\_output\_j\_1}=N_{EZ\_j}*\ln r_{EZ\_j}/r_{elong\_output\_j\_1}$

$r_{elong\_output\_j\_2}=\ln r_{EZ\_output\_j}*R_{prod\_output\_j\_2}$

$t_{resid\_output\_j\_2}=N_{EZ\_j}*\ln r_{EZ\_j}/r_{elong\_output\_j\_2}$

Parameters l_{0EZ}, r_{EZ} were computed by performing a linear fitting on the length of cells at a given time. N_{EZ} was directly given by the model. R_{prod} was computed using the two approaches applied on the length of the virtual root.

S1.E. Theoretical predictions of the Ruler, Timer and Sizer models

To evaluate further the consistency of each model (Ruler, Timer, Sizer) outcomes with those of wild type roots we turned into a theoretical analysis of each model dynamics. Specifically, we searched for the relationships among readily measurable phenotypic traits that each cell differentiation model settles down. Besides the cell length of the EZ cell next to the differentiation zone (l_m), we focused also on additional traits that are

direct and readily measurable quantities in real root cell files: 'number of cells in the EZ (N_{EZ})', 'length of the EZ (L_{EZ})', 'elongation factor (r_{EZ})' and 'length of cells when entering the EZ (l_0)'.

While the two first traits, together with l_m, are dependent on the terminal cell differentiation mechanism to be defined, the two latter ones are not (see section S1.A). We reasoned that each mechanism of cell differentiation can be expected to drive specific relationships between these traits, which in turn should confine their variability. To extract analytically these relationships, we simplified the dynamics to perfect periodic mitotic activity and no cell-to-cell variability between cells in a root file (section S1A).

I. Theoretical relationships: dependence on the cell elongation rate and the meristematic activity.

Herein we calculate how the number of cells in the EZ (N_{EZ}), the length of this zone (L_{EZ}) and the length of EZ cell next to the DZ (l_m) in a root depend on which is the cell elongation rate r_{elong} of its cells and the root's meristematic activity R_{prod}. The relationships found for each model are plotted as continuous lines (gray, black/red and red, for the Ruler, Timer and Sizer models respectively) in the panels depicting data of N_{EZ} vs L_{EZ} , l_m vs $1/r_{EZ}$ and N_{EZ} vs $1/lnr_{EZ}$. All the relationships are computed using the average values of R_{prod}, r_{elong}, l_0 and Threshold corresponding to those values assigned as p (from parameter) in Table EV3. The relationships herein described are depicted with colored continuous lines in Figs. 2,3,6 and Appendix Figs. S8-S10, S13, S16-S18.

1. Ruler model. By definition, in this model the size of the EZ is fixed since it corresponds to the threshold for cell elongation termination, L_{EZ} =L_0 and does not depend on the other traits, nor on the elongation rate, nor on the meristematic activity. Therefore in plots of N_{EZ} vs L_{EZ} the Ruler model predicts a horizontal line of value L_0 (e.g. gray continuous line in Appendix Fig. S9B).

By taking into account that the length of the elongation zone corresponds to the sum of the lengths of all cells within the EZ, which are given by Eq. 6, and that it must be equal to L_0, we obtain

$$L_0 = L_{EZ} = l_0 \frac{1-r_{EZ}^{N_{EZ}}}{1-r_{EZ}} \quad (17)$$

From Eq.17, the number of cells in the EZ, N_{EZ}, can be extracted as a function of L_0, l_0 and r_{EZ}. Yet, this assumes that this number of cells takes continuos values. Since this is not the case, a better approximated equation is

$$N_{EZ} = \frac{1}{2}\frac{ln\left(1+\frac{L_0}{l_0}\left(1-\frac{1}{r_{EZ}}\right)\right)}{lnr_{EZ}} + \frac{1}{2}\frac{ln\left(1-\frac{L_0}{l_0}(1-r_{EZ})\right)}{lnr_{EZ}} \quad (18)$$

which is the relation plotted with a gray continuous line in panels N_{EZ} vs $1/lnr_{EZ}$ (Ruler).

The length of EZ cell next to the DZ can be extracted from Eq. 17 and taking into account that $l_m = l_{i=N_{EZ}-1}$:

$$l_m = l_0 + L_0\left(1 - \frac{1}{r_{EZ}}\right) \quad (19)$$

Eq. 19 is plotted as a continuous gray line in all panels l_m vs $1/r_{EZ}$ (Ruler). All these expressions are computed using the values of l_0 and L_0 in Table EV3 (denoted by p). These three theoretical relationships are a good description of the data obtained from simulations with the Ruler model of roots that only differ in their meristematic activity R_{prod} and their cell elongation rate r_{elong} (Appendix Fig. S9).

Notice this derivation only required assuming Eq. 5 holds, which states that the cell length along the EZ depends exponentially on the cell number from the QC, being a good approximation for real roots (Figs. 1, Appendix Figs. S1, S2).

2. Timer model. By definition, in this model the time spend in the elongation zone is fixed, $T = T_0$. Taking into account that $T = \frac{N_{EZ}}{R_{prod}}$ (section S1.A), the number of cells in the EZ is

$$N_{EZ} = T_0 R_{prod} \quad (20)$$

and is independent of r_{elong}. Eq. 20 using $ln(r_{EZ}) = r_{elong}/R_{prod}$ with r_{elong} and T_0 values given by Table EV3 (values denoted as p) was plotted with a continuous red line in plots N_{EZ} vs $1/lnr_{EZ}$(Timer), whereas Eq. 20 was plotted with the horizontal black continous line in plots N_{EZ} vs $1/lnr_{EZ}$(Timer), using the values denoted as p in Table EV3 for R_{prod} and T_0 .

By imposing $T = T_0$ into Eq. 2, the length of the cell in the DZ is obtained as

$$l_{diff} = l_0 {r_{EZ}}^{N_{EZ}} = l_0 e^{r_{elong}T_0} \quad (21)$$

which is independent of R_{prod}. The length of the EZ cell next to the DZ takes values $l_{diff}/r_{EZ} \leq l_m < l_{diff}$ over time, in the stationary root growth regime, since cells are

discrete units and N_{EZ} takes discrete values. The average l_m is then well approximated by $l_m = l_0 {r_{EZ}}^{N_{EZ}-1/2}$. According to Eq. 21, average l_m can be rewritten as $l_m = \frac{1}{\sqrt{r_{EZ}}} l_0 e^{r_{elong} T_0}$, which specifies the relationship between this average cell length and the elongation factor for distinct values of the meristematic activity R_{prod} of the root (recall that $ln(r_{EZ}) = r_{elong}/R_{prod}$). It is plotted with red continous line in plots N_{EZ} vs $1/lnr_{EZ}$ (Timer) using the fixed values of r_{elong}, T_0 and l_0 in Table EV3 (denoted as p values). By using where Eq. 20, the average l_m can also be rewritten as $l_m = l_0 {r_{EZ}}^{T_0 R_{prod}-1/2}$, which is plotted with black continous line in plots N_{EZ} vs $1/lnr_{EZ}$ (Timer) using the values of R_{prod}, T_0 and l_0 in Table EV3 (denoted as p values). This expression sets the relationship between l_m and the elongation factor for different values of the cell elongation rate.

In plots N_{EZ} vs L_{EZ} (Timer), the theoretical prediction of changes in L_{EZ} driven by distinct values of r_{elong} is a vertical line since N_{EZ} is constant (see Eq. 20). This is plotted with continuous black line at the value of N_{EZ} given by Eq. 20 according to the values in Table EV3. In contrast, the theoretical prediction of changes in L_{EZ} driven by distinct values of R_{prod} can be obtained by adding the cell lengths of all cells in the EZ and using Eqs. 20 and 21. Since cells are discrete units and N_{EZ} takes discrete values, the length of the EZ in the stationary regime takes the values $\sum_{i=0}^{N_{EZ}-1} l_i \leq L_{EZ} < \sum_{i=0}^{N_{EZ}} l_i$. Therefore, the average value of the length of the EZ can be approximated by

$$L_{EZ} = \frac{1}{2}\sum_{i=0}^{N_{EZ}-1} l_i + \frac{1}{2}\sum_{i=0}^{N_{EZ}} l_i = \frac{1}{2} l_0 \frac{1-exp(T_0 r_{elong})}{1-exp\left(\frac{T_0 r_{elong}}{N_{EZ}}\right)} + \frac{1}{2} l_0 \frac{1-exp(T_0 r_{elong})}{exp\left(-\frac{T_0 r_{elong}}{N_{EZ}}\right)-1} \qquad (22)$$

where Eqs. 20 and 21 have been used. Eq. 22 specifies the relationship between the average length of the EZ and its number of cells for distinct values of the meristematic activity R_{prod}. It is plotted with a red continous line in plots N_{EZ} vs L_{EZ} (Timer) using the values of r_{elong}, T_0 and l_0 given by Table EV3 (denoted as p values).

These theoretical relationships are a good description of the numerical data obtained from simulations of the Timer model of many roots that are all the equal except for the meristematic activity R_{prod} or except for the cell elongation rate r_{elong} that each root has (Appendix Fig. S10). When roots differ in both R_{prod} and r_{elong}, mixed relationships arise for simulations of the Timer model (Appendix Fig. S9).

3. Sizer model. In this case, the cell length of cells in the DZ is fixed as

$$l_{diff} = l_{diff\,0} \quad (23)$$

Hence, it is independent of r_{EZ}. The average l_m is then well approximated by $l_m = l_0 r_{EZ}{}^{N_{EZ}-1/2}$and hence $l_m = l_{diff\,0} r_{EZ}{}^{-1/2}$, which is plotted with a red continous line in plots l_m vs $1/r_{EZ}$ (Sizer) by using the value of $l_{diff\,0}$ in Table EV3.

By setting Eq. 23 into Eq. 2 for $l_{diff} = l_{i=N_{EZ}}$ (i.e. $l_{diff} = l_0 r_{EZ}{}^{N_{EZ}}$) N_{EZ} becomes:

$$N_{EZ} = \frac{ln(l_{diff\,0}/l_0)}{lnr_{EZ}} \quad (24)$$

which is plotted with a continuous red line in plots N_{EZ} vs $1/lnr_{EZ}$(Sizer) using the values of $l_{diff\,0}$ and l_0 in Table EV3.

The length of the EZ in the Sizer model is obtained by re-writing the elongation factor r_{EZ} in Eq. 17 in terms of N_{EZ} using Eq.24:

$$L_{EZ} = l_0 \frac{1-l_{diff\,0}/l_0}{1-(l_{diff\,0}/l_0)^{\frac{1}{N_{EZ}}}} \quad (25)$$

These theoretical relationships are a good description of the data obtained from in silico Sizer model simulations of roots that are equal except for their values of meristematic activity R_{prod} and the cell elongation rate r_{elong} that each root has (Appendix Fig. S9). Notice this derivation only required assuming Eq. 2 holds, which states that the cell length along the EZ depends exponentially on the cell number from the QC, which is a good approximation in real roots (Fig. 1, Appendix Figs. S1, S2). In addition, analogous relationships would be found if it were the fold-change in cell length along the whole EZ (i.e. the ratio between the cell length at existing the EZ and the length when entering it), the one setting the threshold for cell elongation termination.

These results show that the relationship between the length and the number of cells in the EZ depends on the model. They also show that the relationships between the length of mature cells or the number of cells in the EZ with the elongation factor is also distinct among models, depending differently on the cell elongation rate and differentiation thresholds.

II. Theoretical relationships: dependence on the threshold that sets cell elongation termination.

Herein we consider that all parameters except for the differentiation threshold (i.e. the threshold value that sets termination of cell elongation) are the same between all roots.

Therefore, parameters R_{prod}, r_{elong} and l_0 are the same for all roots. Accordingly, the elongation factor is also the same for all roots and corresponds to $r_{EZ} = e^{\frac{r_{elong}}{R_{prod}}}$. In this scenario, the length of the elongation zone in terms of the number of cells in this zone is then given simply by Eq. 6 and is the same for all three (Ruler, Timer and Sizer) models. This is the blue line depicted in all plots of N_{EZ} vs L_{EZ} using the values of R_{prod}, r_{elong}, l_0 corresponding to those values assigned as p (from parameter) in Table EV3. In Appendix Fig. S8 this scenario is numerically evaluated through simulations of roots which are all the same except for the threshold value at which they terminate elongation. Numerical results confirm all models conform to this theoretical prediction (Appendix Fig. S8).

S1.F. Computations used in Fig.4.

- Fig.4C. The production rate is proportional to $1/\ln(r_{EZ})$, so two fold changes in R_{prod} can be simulated by applying two fold changes to the value of $1/\ln(r_{EZ})$. For the case of real R_{prod}, the real values extracted from all 8-day-old plants analyzed in this study (Approach 1+ Approach 2) (white diamonds) were used (white). For the case of $R_{prod}/2$ and $2R_{prod}$, we used real values of $1/(2\ln(r_{EZ}))$ and $2/\ln(r_{EZ})$, respectively, and we constructed N_{EZ} for each plant as the slope multiplied with the real value of $1/(2\ln(r_{EZ}))$ and $2/\ln(r_{EZ})$, respectively, plus a random number between -1.5 and 1,5.
- Fig. 4D: For the case of real R_{prod}, we maintained the real values for $1/\ln(r_{EZ})$ and shuffled the real values of N_{EZ}. In this way, we maintained the same range of values, but we disrupted the meristem-elongation correlation. We used all 8-day-old data (Approach 1+ Approach 2). For the case of $R_{prod}/2$ and $2R_{prod}$, we used real values of $1/(2\ln(r_{EZ}))$ and $2/\ln(r_{EZ})$, respectively, and we constructed N_{EZ} for each plant by shuffling the real values of N_{EZ}.
- Fig. 4E-H Simulated l_{diff} and R_{growth} distributions were generated using equations (5) and (4) applied to the values from Fig. 4C (correlation) and Fig. 4D (no correlation) and the real values for $l_{0EZ.}$

S2. Supplementary Notes.

S2.A. On the results concerning the method to set the boundary between the MZ and EZ. The results showed that our definition of the meristematic zone is consistent with the apical epidermal meristem (Table EV2) and shows that the epidermal MZ reaches the stationary regime at 6 days after germination, consistent with similar results obtained in cortical cells (Moubayidin *et al*, 2010; Dello Ioio *et al*, 2008). Our results show that on the stationary regime the meristem has 25 epidermal cells and 31 cortical cells on average, meanwhile the elongation zone has an average of 14 epidermal and 11 cortical cells.

Our results for the cortex tissue are in agreement with previously reported quantifications (Dello Ioio *et al*, 2008; Moubayidin *et al*, 2010; Hacham *et al*, 2011; Mähönen *et al*, 2014). These quantifications were made through measuring the length of the cortical cells starting from QC until the first rapidly elongated cell. Mahonen et al considered the upper boundary of the elongation zone where the first root hair appears and the protoxylem starts differentiating The number of cells in the meristem of the epidermal file computed through our quantitative method is slightly smaller (25 versus 32) than the one previously measured by finding the cell which is two-fold change larger than the previous one (González-García *et al*, 2011a). This may be due to a smoother length transition of cells from the meristem to the elongation zone in the epidermal cell file, which makes it more difficult to define the boundary. This inconvenient is overcome using our approach.

S2.B. On the agreement of extracted average quantitative values of Arabidopsis roots with previously reported data. The results of the inference for wild type plant roots show that the meristem epidermal cells divide at a rate of 1.4±0.4 $days^{-1}$ per cell in the stationary regime, and 1.3±0.6 $days^{-1}$ per cell in cortex files. In the EZ, epidermal cells elongate at a rate of 0.28±0.8 h^{-1} during 10±.4 h, and cortex cells elongate at a rate of 0.42±0.16 h^{-1} during 8±3 h. Using a similar approach but applied on a fitting on several plants altogether, with the cell position axis converted in a time axis and with a different method to define the MZ, (Cole *et al*, 2014)obtained for the cortex a division rate in the meristem of 1.15 $days^{-1}$ per cell, an elongation rate of 0.41 h^{-1} and a residence time in the EZ of 7 h. Also in agreement with our measurements, previous studies based on kinematic methods have obtained cortical cell division rates of 1±0.2 cells/(cells*day) (Beemster & Baskin, 1998; Rymen *et al*; Beemster & Baskin, 2000) and a residence time in the EZ of 8 h (Baskin *et al*, 1995; Beemster & Baskin, 1998,

2000). Recently, clonal analysis and video tracking have also shown that cortex cells stay 6-8h in the EZ (Mähönen *et al*, 2014). Therefore, we obtained a reliable, easy and complete extraction of several cellular dynamical traits from spatial root measurements.

S3. Program Codes

S3.A. Code for the automated fitting of individual roots

Here below the code run in Octave version 4.2.1 for the automated fitting on individual roots is detailed:

```
*************************
function Automated fitting



clear, clc, close all, format compact, format long
[FileName,PathName,FilterIndex] = uigetfile('.xls', 'Choose the excel file');
if ischar(FileName) == 0
  if FileName == 0 && PathName == 0
    return
  end
end

%import the logarithm of the data from sheet u into 'data' array
for u=1:8
  data=zeros(1,1);
  data=log(xlsread(strcat(FileName),u,'B2:bm100'));
%y=vector containing the data extracted from the Excel file
qwrt=zeros(16,1);
A=zeros(16,1);
B=zeros(50,1);
%assign a column to y and eliminate the elements equal to NaN
for i=1:size(data,2)
    y=zeros(1,1);
    for l=1:size(data,1)
      if isnan(data(l,i))==0
        y(l,1)=data(l,i);
      end
    end
        clear p
    clear v
    clear RSS1
    clear TSS1
    clear RSS2
    clear TSS2
    clear Rsq1
    clear Rsq2
    clear R
%Generate the position vector x (1,2,3,.....). Fill the array x with consecutive
numbers until the last row occupied
```

```
   x=zeros(size(y,1),1);
   x(1,1)=0;
for j=1:size(y,1)-1
  x(j+1,1)=x(j,1)+1;
end
   count=zeros(1,1);
   R=zeros(1,1);
   res1=zeros(1,1);
   %We consider MZ to have at least 5 cells and EZ to have at least 3 cells, so we
impose z>=5 and z<size(y,1)-3
   %The linear fit (polyfit)is applied to all the points from 1 to z and from z+1 to

   %ruz=elongation factor in MZ, represents the exp from the slope of the fit for the
first z points
   %l0uz= exp from the intercept of the fit for the first z points
   %l0tz=exp from the intercept of the fit for or the points from z+1 to the end
   %rtz=elongation factor in EZ, represents the exp from the slope of the fit for the
points from z+1 to the end
   %z=the number of cells in the MZ
   %NEZ= the number of cells in EZ
   %fitting=custom function to create a linear fit of the form y=slope*x+intercept
   for z=5:size(y,1)-3
       p=polyfit(x(1:z,1),y(1:z,1),1);
       v=polyfit(x(1:size(y,1)-z+1,1),y(z:size(y,1),1),1);
       ruz(z)=exp(abs(p(1,1)));
       l0uz(z)=exp(abs(p(1,2)));
       rtz(z)=exp(abs(v(1,1)));
       l0tz(z)=exp(abs(v(1,2)));
       NEZ(z)=size(y,1)-z;
       yval=exp(y);
       LMZ(z)=sum(yval(1:z,1),1);
       LEZ(z)=sum(yval(z+1:size(yval,1),1),1);
       Ltot(z)=sum(yval,1);
       fit1=fitting(x(1:z,1),p(1,1),p(1,2));
       fit2=fitting(x(1:size(y,1)-z+1,1),v(1,1),v(1,2));
       RSS1(z)=sum((y(1:z,1)-fit1(1:z,1)).^2);
       TSS1(z)=sum((y(1:z,1)-mean(y(1:z,1))).^2);
       Rsq1(z)=1-RSS1(z)/TSS1(z); %R squared, shows how good is the fit for MZ
       RSS2(z)=sum((y(z:size(y,1),1)-fit2).^2);
       TSS2(z)=sum((y(z:size(y,1),1)-mean(y(z:size(y,1),1))).^2);
       Rsq2(z)=1-RSS2(z)/TSS2(z); %R squared, shows how good is the fit for EZ
       R(z)=sqrt(abs(Rsq1(z)+Rsq2(z))); %is maximum when both fits are good
     %count=control parameter; it prevents the finding of multiple intersection
points
     if count~=0
        break
     end
```

```
      prueba=0; %prueba=control parameter;
     if (l0uz(z)*ruz(z)^(z-2))/(l0tz(z)/rtz(z))>1 &&(l0uz(z)*ruz(z)^(z-1)/l0tz(z)<=1)&& (l0uz(z)*ruz(z)^(z))/(l0tz(z)*rtz(z))<1
        prueba=1;
        res1=z;
        res2=NEZ(z);
        res3=ruz(z);
        res4=l0uz(z);
        res5=rtz(z);
        res6=l0tz(z);
        res7=l0uz(z)*(1-ruz(z)^(z))/(1-ruz(z));

        res8=l0tz(z)*(1-rtz(z)^(NEZ(z)))/(1-rtz(z))-l0tz(z);
        res9=res7+res8;
        yval=exp(y);
        res10=sum(yval(1:z));
        res11=sum(yval(z+1:size(y,1),1),1);
        res12=sum(yval,1);
        res13=Rsq1(z);
        res14=Rsq2(z);

       %we impose that the no of cells in EZ is smaller than the no of cells in MZ
        if res1<res2
           prueba=0;
        end
        crit=1; %parameter that contains the number of the criterion applied
     end
    count=count+prueba;
 end

 if count==0
    clear p
    clear v
    clear RSS1
    clear TSS1
    clear RSS2
    clear TSS2
    clear Rsq1
    clear Rsq2
    clear R

    for z=5:size(y,1)-3
        p=polyfit(x(1:z,1),y(1:z,1),1);
        v=polyfit(x(1:size(y,1)-z+1,1),y(z:size(y,1),1),1);
        ruz(z)=exp(abs(p(1,1)));
        l0uz(z)=exp(abs(p(1,2)));
        rtz(z)=exp(abs(v(1,1)));
        l0tz(z)=exp(abs(v(1,2)));
        NEZ(z)=size(y,1)-z;
```

```
        yval=exp(y);
        LMZ(z)=sum(yval(1:z,1),1);
        LEZ(z)=sum(yval(z+1:size(yval,1),1),1);
        Ltot(z)=sum(yval,1);


        fit1=fitting(x(1:z,1),p(1,1),p(1,2));
        fit2=fitting(x(1:size(y,1)-z+1,1),v(1,1),v(1,2));
        RSS1(z)=sum((y(1:z,1)-fit1(1:z,1)).^2);
        TSS1(z)=sum((y(1:z,1)-mean(y(1:z,1))).^2);
        Rsq1(z)=1-RSS1(z)/TSS1(z); %R squared, shows how good is the MZ fit
        RSS2(z)=sum((y(z:size(y,1),1)-fit2).^2);
        TSS2(z)=sum((y(z:size(y,1),1)-mean(y(z:size(y,1),1))).^2);
        Rsq2(z)=1-RSS2(z)/TSS2(z); %R squared, shows how good is the EZ fit
        R(z)=sqrt(abs(Rsq1(z)+Rsq2(z))); %is maximum when both fits are good

      %count=control parameter; it prevents the finding of multiple intersection
points
      if count~=0
         break
      end


      prueba=0; %prueba=control parameter
     if (l0uz(z)*ruz(z)^(z-2))/(l0tz(z)/rtz(z))>1 &&
(l0uz(z)*ruz(z)^(z))/(l0tz(z)*rtz(z))<1
        prueba=1;
        res1=z;
        res2=NEZ(z);
        res3=ruz(z);
        res4=l0uz(z);
        res5=rtz(z);
        res6=l0tz(z);
        res7=l0uz(z)*(1-ruz(z)^(z))/(1-ruz(z));
        res8=l0tz(z)*(1-rtz(z)^(NEZ(z)))/(1-rtz(z))-l0tz(z);
        res9=res7+res8;
        yval=exp(y);
        res10=sum(yval(1:z));
        res11=sum(yval(z+1:size(y,1),1),1);
        res12=sum(yval,1);
        res13=Rsq1(z);
        res14=Rsq2(z);

       %we impose that the no of cells in EZ is smaller than the no of cells in MZ
        if res1<res2
           prueba=0;
        end
        crit=3;
     end
```

```
    count=count+prueba;
    end
  end

 %when count is 0 after the previous loop, criteria 1 and 3 were not fulfilled, so we
pass to criterion 2, which searches for the maximum value of R and sets the value
for crit to 2
  while  count==0
         ind=find(R>=max(R));
         res1=ind;
         res2=NEZ(ind);
         res3=ruz(ind);
         res4=l0uz(ind);
         res5=rtz(ind)
         res6=l0tz(ind);
         res7=l0uz(ind)*(1-ruz(ind)^(ind))/(1-ruz(ind));
         res8=l0tz(ind)*(1-rtz(ind)^(NEZ(ind)+1))/(1-rtz(ind));
         res9=res7+res8;
         yval=exp(y);
         res10=sum(yval(1:z));
         res11=sum(yval(z+1:size(y,1),1),1);
         res12=sum(yval,1);
         res14=Rsq2(ind);
         res13=Rsq1(ind);
         crit=2;
         count=1;
  end

 %ajusteMZ= creates a fit of the form: l0*r^i
 ajusteMZ=zeros(size(y(1:res1),1),1);
 ajusteMZ(1,1)=l0uz(res1);
 ajusteEZ=zeros(size(y,1)-res1,1);
 ajusteEZ(1,1)=l0tz(res1)*rtz(res1);
 for q=1:res1-1
    ajusteMZ(q+1)=ajusteMZ(q)*ruz(res1);
 end
 for w=1:size(y,1)-res1-1
    ajusteEZ(w+1)=ajusteEZ(w)*rtz(res1);
 end
 %B=matrix which contains one column with both fits, for MZ and EZ
 B(1,i)=i;
 B(2:size(y,1)+1,i)=[ajusteMZ;ajusteEZ];
 % Constraint: if the diff between rMZ and rEZ is very small, than only one zone
should be considered with only one fit for all the points
   if abs(res3-res5)<=0.08
      pp=polyfit(x,y,1);
      res1=size(y,1);
      res2=0;
      res3=exp(abs(pp(1,1)));
```

```
      res4=exp(abs(pp(1,2)));
      res5=0;
      res6=0;
      res7=res4*(1-res3^res1)/(1-res3);
      res8=0;
      res9=res7+res8;
      yval=exp(y);
      res10=sum(yval,1);
      res11=0;
      res12=0;
      fit3=fitting(x(1:size(y,1),1),abs(pp(1,1)),abs(pp(1,2)));
      RSS1=sum((y(1:size(y,1),1)-fit3(1:size(y,1),1)).^2);
      TSS1=sum((y(1:size(y,1),1)-mean(y(1:size(y,1),1))).^2);
      Rsq1=1-RSS1/TSS1;
      res13=Rsq1;
      res14=0;
      ajusteMZ=zeros(size(y,1),1);
      ajusteMZ(1,1)=res4;
       for q=1:res1-1
          ajusteMZ(q+1)=ajusteMZ(q)*res3;
       end
      B(2:size(y,1)+1,i)=ajusteMZ;
  end

qwrt=[i;crit;res1;res2;res3;res4;res5;res6;res7;res8;res9;res10;res11;res12;res13;
res14]; %qwrt=matrix containing the extracted grwht parameters, where each
column corresponds to one plant

 A=[A qwrt]; %A=matrix containing the resulting growth parameters for all the
plants. It¥s different for each sheet

end
  xlswrite(strcat('avg_results_actualized7_4_',FileName),A,u);
  xlswrite(strcat('avg_results_actualized7_4_',FileName,'_data_fit_v7_4.xls'),B,u);
end
*********************
function [fit] = fitting(x,p1,p2 )%creates the linear fit y=slope*x+intercept
fit=p1*x+p2;
end
*********************
```

References

Band LR, Úbeda-Tomás S, Dyson RJ, Middleton AM, Hodgman TC, Owen MR, Jensen OE, Bennett MJ & King JR (2012) Growth-induced hormone dilution can explain the dynamics of plant root cell elongation. *Proc. Natl. Acad. Sci. U. S. A.* **109:** 7577–7582

Baskin TI, Cork a., Williamson RE & Gorst JR (1995) STUNTED PLANT 1, A Gene

Required for Expansion in Rapidly Elongating but Not in Dividing Cells and Mediating Root Growth Responses to Applied Cytokinin. *Plant Physiol.* **107:** 233–243
Beemster GT & Baskin TI (1998) Analysis of cell division and elongation underlying the developmental acceleration of root growth in Arabidopsis thaliana. *Plant Physiol.* **116:** 1515–1526
Beemster GT & Baskin TI (2000) Stunted plant 1 mediates effects of cytokinin, but not of auxin, on cell division and expansion in the root of Arabidopsis. *Plant Physiol.* **124:** 1718–1727
Cole RA, Mcinally SA & Fowler JE (2014) Developmentally distinct activities of the exocyst enable rapid cell elongation and determine meristem size during primary root growth in Arabidopsis. **:** 1–20
Eaton JW, Bateman D, Hauberg S & Wehbring R GNU Octave version 4.0.0 manual: a high-level interactive language for numerical computations.
González-García M-P, Vilarrasa-Blasi J, Zhiponova M, Divol F, Mora-García S, Russinova E & Caño-Delgado AI (2011a) Brassinosteroids control meristem size by promoting cell cycle progression in Arabidopsis roots. *Development* **138:** 849–859
González-García M-P, Vilarrasa-Blasi J, Zhiponova M, Divol F, Mora-García S, Russinova E & Caño-Delgado AI (2011b) Brassinosteroids control meristem size by promoting cell cycle progression in Arabidopsis roots. *Development* **138:** 849–59
Hacham Y, Holland N, Butterfield C, Ubeda-Tomas S, Chory J, Savaldi-Goldstein S, Bennett MJ, Chory J & Savaldi-Goldstein S (2011) Brassinosteroid perception in the epidermis controls root meristem size. *Development* **138:** 839–848
Dello Ioio R, Nakamura K, Moubayidin L, Perilli S, Taniguchi M, Morita MT, Aoyama T, Costantino P & Sabatini S (2008) A genetic framework for the control of cell division and differentiation in the root meristem. *Science* **322:** 1380–1384
Ivanov VB & Dubrovsky JG (1997) Estimation of the Cell-Cycle Duration in the Root Apical Meristem: A Model of Linkage between Cell-Cycle Duration, Rate of Cell Production, and Rate of Root Growth. *Int. J. Plant Sci.* **158:** 757
Kang YH, Breda A & Hardtke CS (2017) Brassinosteroid signaling directs formative cell divisions and protophloem differentiation in *Arabidopsis* root meristems. *Development* **144:** 272–280
Mähönen AP, Tusscher K Ten, Siligato R, Smetana O, Díaz-Triviño S, Salojärvi J, Wachsman G, Prasad K, Heidstra R & Scheres B (2014) PLETHORA gradient formation mechanism separates auxin responses. *Nature*
Moubayidin L, Perilli S, Dello Ioio R, Di Mambro R, Costantino P & Sabatini S (2010) The rate of cell differentiation controls the Arabidopsis root meristem growth phase. *Curr. Biol.* **20:** 1138–1143
R Core Team (2016). R: A language and environment for statistical computing.
Rymen B, Coppens F, Dhondt S, Fiorani F & Beemster GTS Chapter 14 Kinematic Analysis of Cell Division and Expansion. *Methods Mol. Biol.***:** 203–227

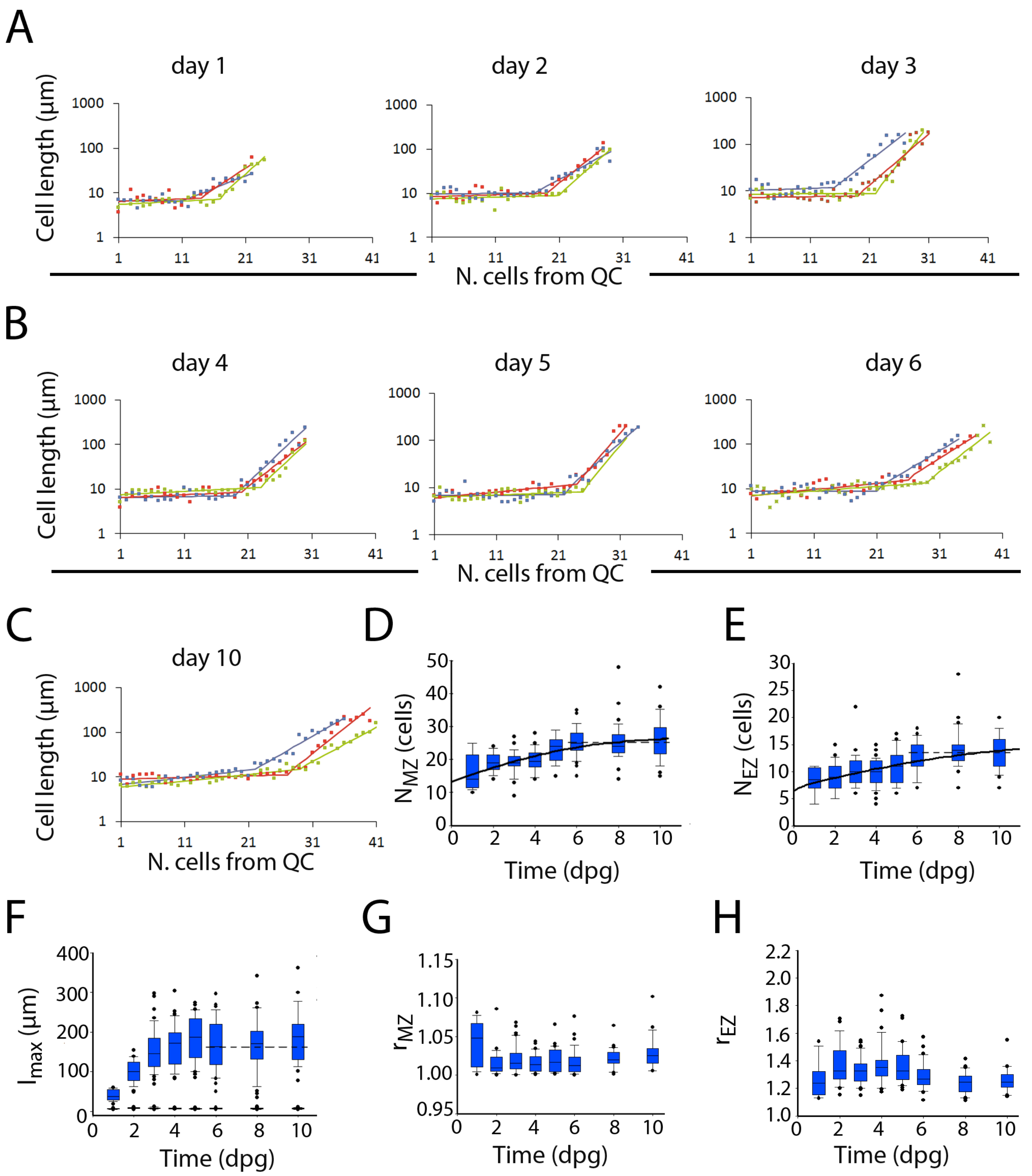

Appendix Figure S1. Root growth parameters for WT epidermal cells

A-C. Length of epidermal cells from 1-5, 8 and 10-day-old (day post germination, dpg) Col-0 WT plants, as a function of the cell number position from the QC, for 3 different individual plants (colored symbols). Logarithmic scale for cell length axis is used. Straight lines stand for the fitted curves in each zone assuming an exponential behavior in a linear scale (using the Method described in Appendix S1B and with the program code in S3A) and their connection. The slope of each fitted curve sets the growth parameter r_{MZ} for the meristematic zone and r_{EZ} for the elongation zone (the slope corresponds to the logarithm of this parameter). We name r_{EZ} as the elongation factor in the elongation zone. **D-H**. Number of cells in the meristem (D) and the elongation zone (E), length of the EZ cell closest to the DZ, (F) and growth parameters (G,H) over time. In D-F the stationary regime is reached at day 6 dpg. Black dashed lines represent the average value characterizing the steady state, computed from the values at days 6,8 and 10 dpg. Black continuous lines mark the tendency. For all the boxplots, boxes represent the interquartile range (25th-75th percentiles, with the median indicated by the black horizontal line and the average indicated by the dashed black line) of the distribution, whiskers extend to the 10th and 90th percentiles and outliers are represented by black dots. The number of plants is detailed in Table EV1. Panels A-C depict the Col-0 epidermal data in Dataset EV1. Panels D-H depict the Col-0 epidermal data in Dataset EV2. Day 6-10 data are used in Figure 2 and Table EV2.

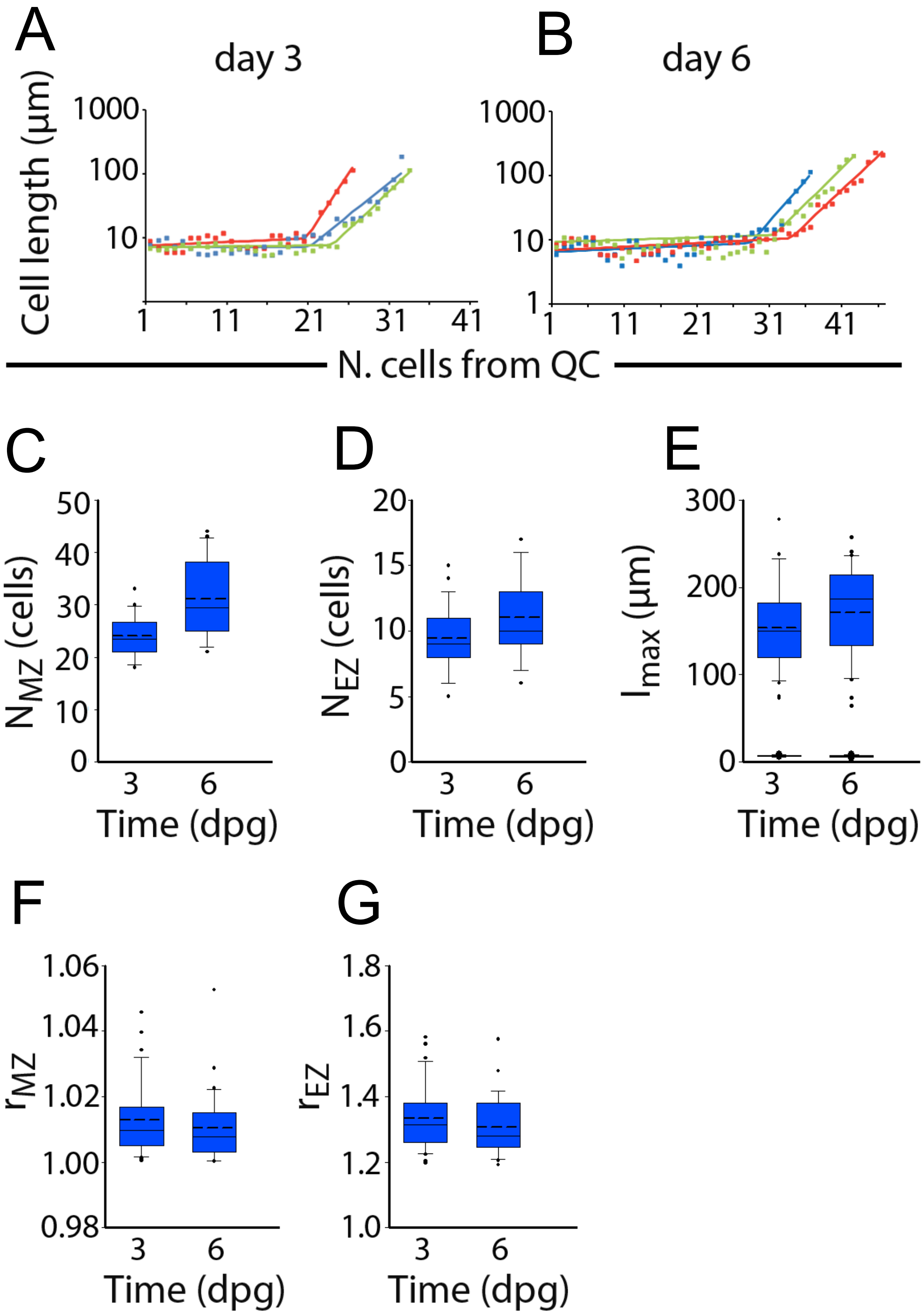

Appendix Figure S2. Root growth parameters for WT cortical cells

A,B. Length of cortical cells from 3 (A) and 6-day-old (B) WT plants, as a function of the cell number position from the QC, for 3 different individual plants (colored symbols). Logarithmic scale for cell length axis is used. Straight lines stand for the fitted curves in each zone assuming an exponential behavior in a linear scale and their connection. The slope of each fitted curve sets the growth parameter r_{MZ} for the meristematic zone and r_{EZ} for the elongation zone (the slope corresponds to the logarithm of this parameter). We name r_{EZ} as the elongation factor in the elongation zone. Note the similarity in behavior between cortical and epidermal cells (Fig. S1). **C-G.** 3 and 6 day-old WT plants boxplot distributions for the number of cells in the meristem (C) and the elongation zone (D), length of the EZ cell closest to the DZ (E) and growth parameters in the meristem (F) and the elongation zone (G), all for cortical cells. Boxplots represented as in Appendix Fig. S1. Panels A-B depict the Col-0 cortex data in Dataset EV1. Panels C-G depict the Col-0 cortex data in Dataset EV2.

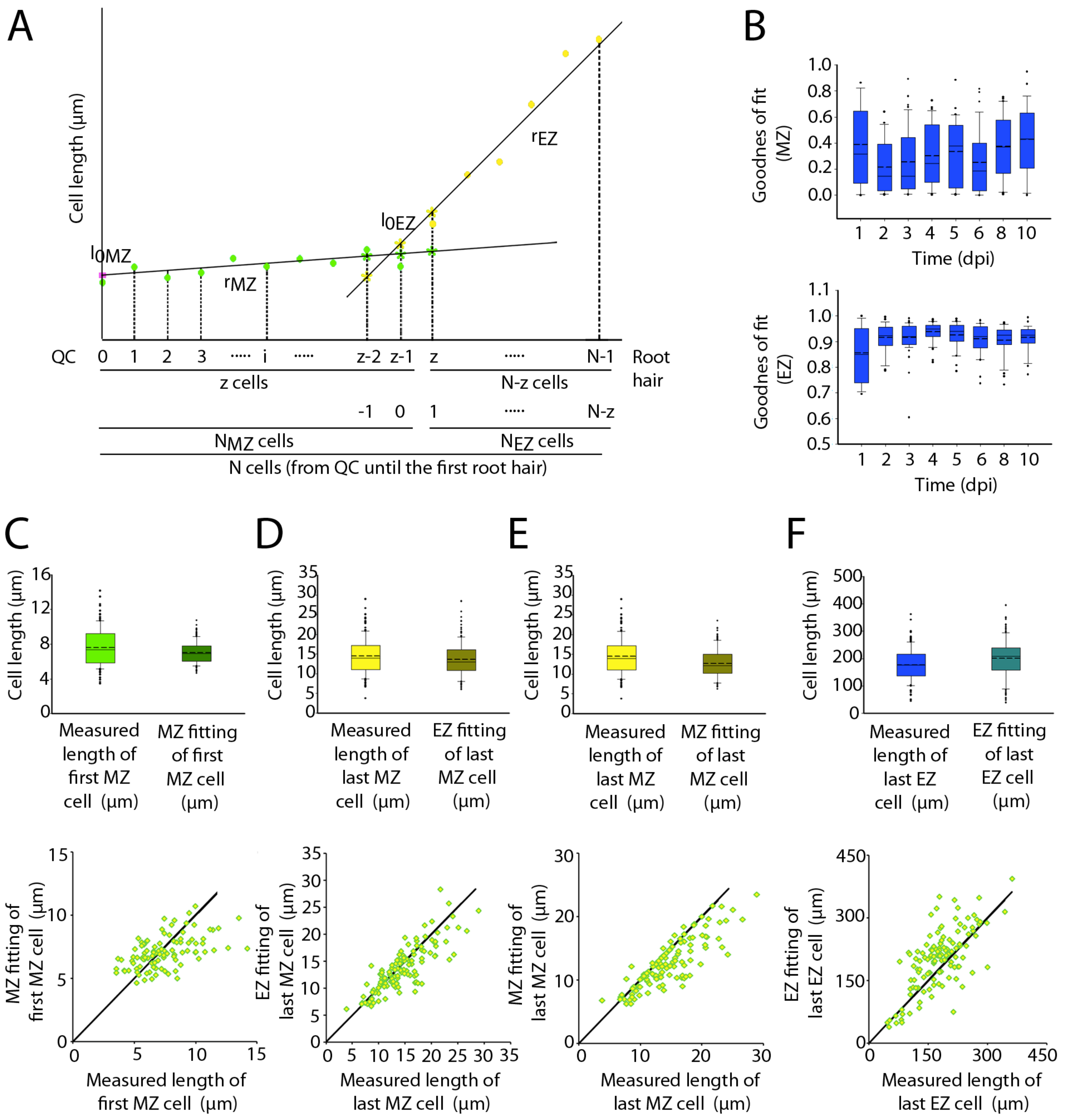

Appendix Figure S3. Validation of the goodness of fit to epidermal WT data

A. Visualization of the method used to extract the cell number of each zone and the growth parameters. This method is described in Appendix Text S1B and the program code is provided in Appendix Text S3A. The plot shows the cell length as a function of cell position, starting from QC, until the appearance of the first root hair (N cells), in logarithmic scale. Based on the exponential behavior of measured cell lengths, a separation between the division zone (green dots) and elongation zone (yellow dots) can be made by applying iteratively two independent linear regressions on the logarithm of the cell lengths as depicted by the straight lines. One of the regressions is on the first z points and the second one is on the last N-z points. The selected pair of fittings depicted intersect between position z-2 and position z-1, and the start of the elongation zone is set in position z. Each linear regression is characterized by its own slope (r_{MZ} and r_{EZ}), and intersection (l_{0MZ} and l_{0EZ}). Green and yellow stars represent the evaluation points for the intersection condition. **B**. Goodness of linear regressions for the pairs of selected fittings: coefficient of determination for the

(continues in next page)

continuation of Appendix Figure S3 caption

linear regressions in the meristematic zone (upper panel) and elongation zone (lower panel) for each day analyzed. In the meristematic zone, the coefficient of determination is much smaller than in the elongation zone, probably because the length of cells changes slightly with position from QC. **C-F.** Each panel shows the measured values and values extracted from linear fittings for the length of the first cell in the meristem (C), the length of the last meristematic cell (D and E) and the length of the last elongated cell (F). Upper panels: For each panel, boxplots of the measured values (left) and of the values expected from fittings (right). Lower panels: The same data as in the corresponding upper panel but represented for each plant. Each dot is the measured value represented against the value expected from fitting for each plant. The straight line corresponds to the curve measured=expected. If linear fittings fit appropriately each zone, we expect the dots to be close to the straight line. In C, the value of the length of the first meristematic cell extracted from linear fitting is the intercept of the MZ fitting (pink square in A). In D, the EZ fitting of last MZ cell corresponds to $l_{0EZ,j}$ where the subindex j denotes the plant. In E, the MZ fitting of last MZ cell corresponds to $l_{0MZ,j}r_{MZ,j}^{NMZ,j-1}$. In F, the EZ fitting of last EZ cell corresponds $l_{0EZ,j}r_{EZ,j}^{NEZ,j}$. See Appendix Text S1C for more details. Boxplots represented as in Appendix Fig. S1. The data are those from epidermis Col-0 WT day 6,8,10 (Approach 1) and day 8 (Approach2) in Dataset EV2, as in Table EV2 and in Figure 2.

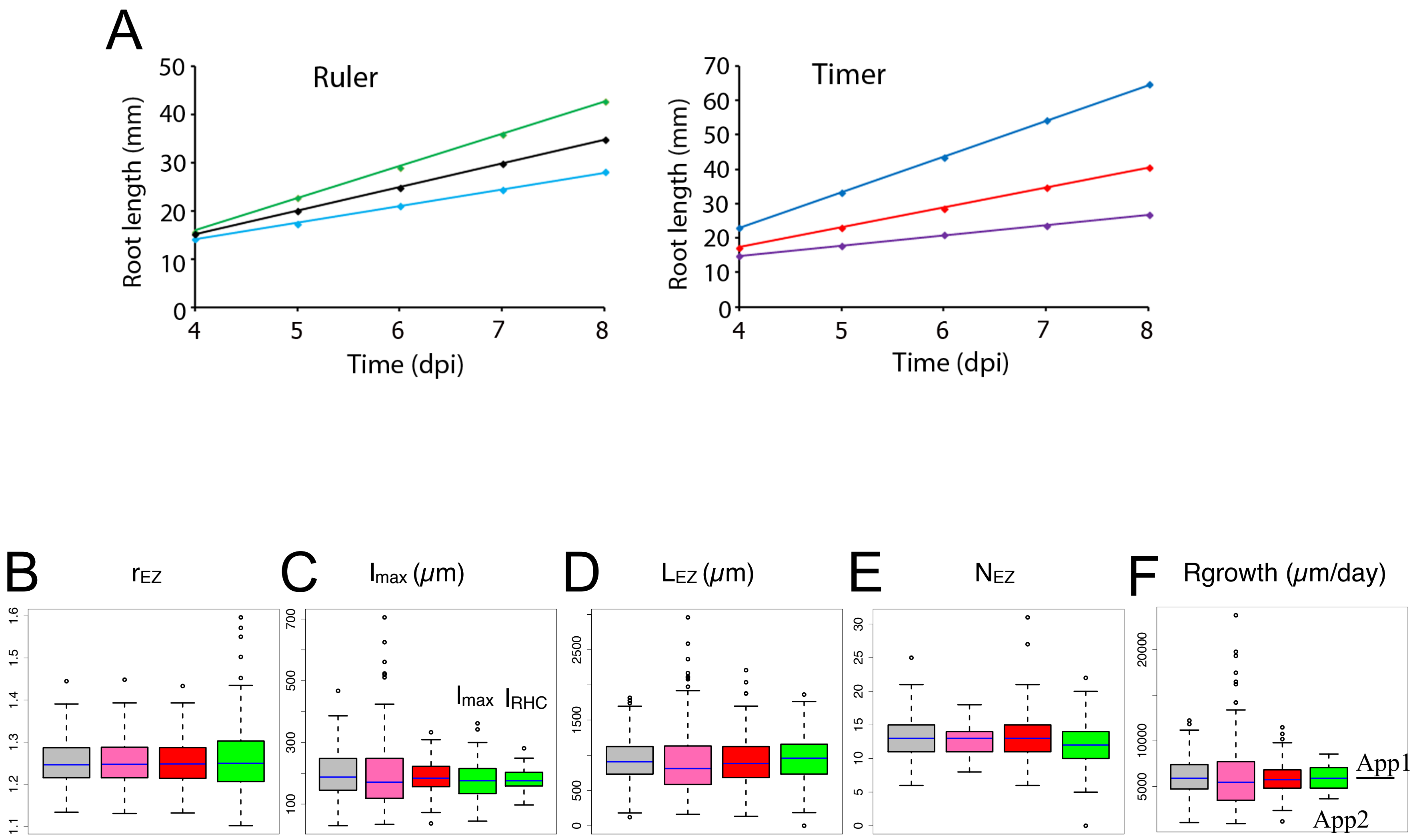

Appendix Figure S4. Root growth in Ruler, Timer and Sizer models and its comparison with WT epidermal data. A. Computational root length (computed as the sum of the lengths of all differentiated cells) at four time points corresponding to the stationary growth of the simulated root, in Ruler and Timer models. Colors stand for three simulated roots. The analogous data for the Sizer model are depicted in Fig.1C(bottom). Parameter values as in Table EV3 (Generic case). **B-F**. Boxplots for phenotypic traits: (B) elongation factor r_{EZ} , (C) length of the EZ cell closest to the DZ l_{max}, (D) length of the elongation zone L_{EZ}, (E) number of cells in the elongation zone N_{EZ}, and (F) root growth rate. In each boxplot, data from Ruler (gray), Timer (pink) and Sizer (red) models (n=121 each) are compared with data from WT epidermis (green) (n=122, corresponding to day 6,8,10 (Approach 1) and day 8 (Approach 2) in Dataset EV2). In C, the Col-0 WT measured data of the cell length of the first root hair cell is also depicted (l_{RHC}, n=42). No statistical significant difference is found between l_{RHC} and l_{max} (p-value=0.7029 for Wilcoxon rank sum test). In F, the WT root growth rate measured through Approach1 (App1, $R_{growth\ exp\ 1}$, which corresponds to a single average value on n=20 roots) and Approach 2 (App2, $R_{growth\ exp\ 2}$, n=22 roots) is given (see Materials and Methods for a description of each Approach and Table EV7). p-values for Wilcoxon rank sum test between each model data for n=1000 and the WT epidermal data (n=122) are detailed in Table EV4 (all are p>0.01). In B-F, for each model, the simulated roots and cells differ in the threshold value for cell elongation termination, the cell elongation rates, the meristematic activities and the initial cell length as defined in Methods (parameter values detailed in Table EV3- WT epidermis case). The parameter values are the same for the three models except for the threshold for cell elongation termination, which is specific of each model and has relative variability of 35% (Ruler), 7% (Timer) and 26% (Sizer). The Timer model is more sensitive to the variability, driving less reliable root growth rates. The relationships between traits that these same simulations exhibit are depicted in Fig. 2. Boxplots represented as in Fig.2.

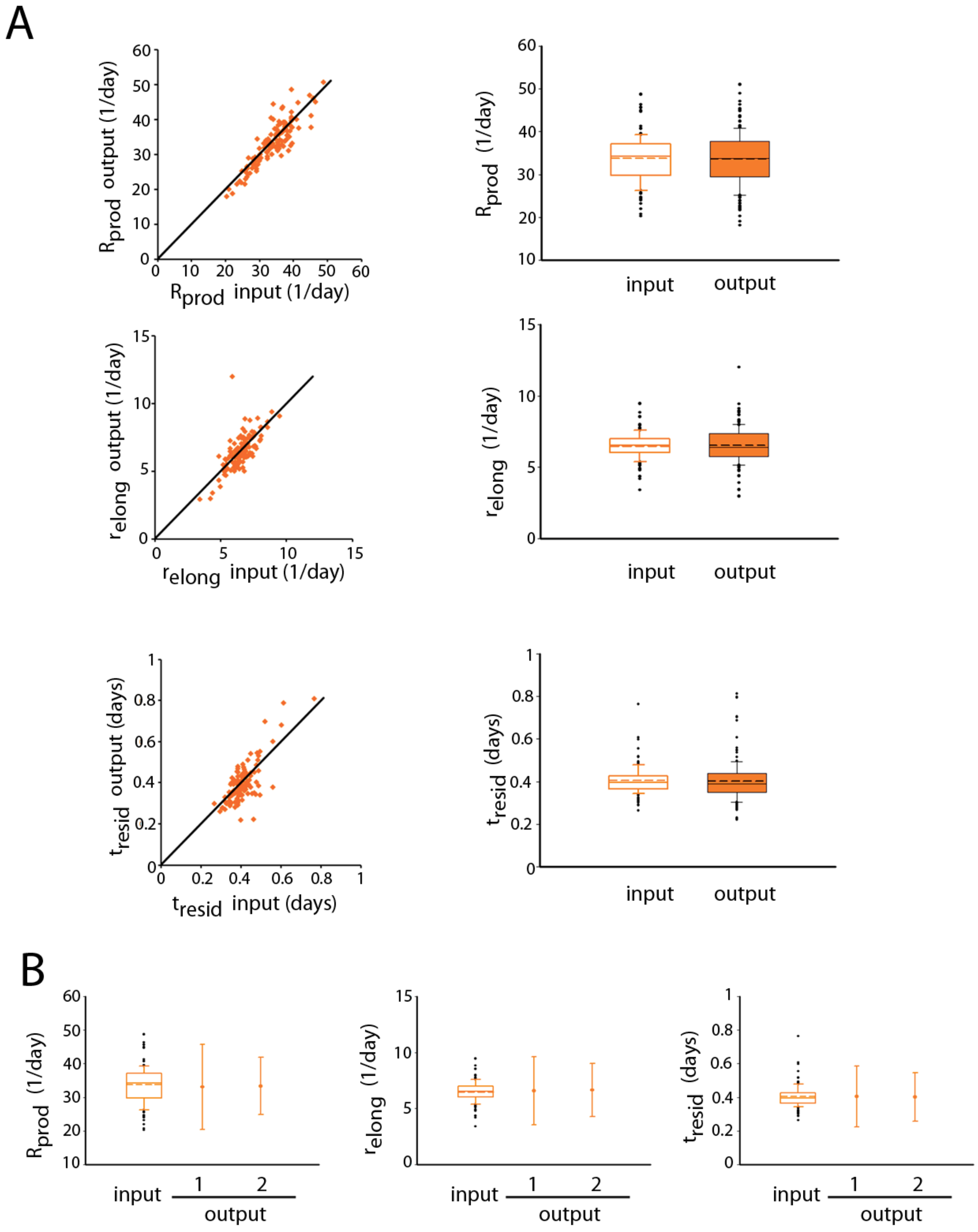

Appendix Figure S5. Validation of the methods used to infer dynamical information, using the computational data of a Sizer model. We analyzed the computational data obtained from simulations of a Sizer model through the methods applied on real WT roots to extract dynamical information of them (formulae in Table EV2, and methods detailed in Appendix Text S1A,D). The advantage of applying these methods on the simulated computational data is that we can compare the extracted dynamical information (output) to the real one of the computational model (input), and hence evaluate whether the methods are providing accurate dynamical information. The only difference being that the models do not contain a MZ and that herein the length of mature cells in the DZ is used (instead of the length of the EZ cell closest to the DZ). Details on the computation of input and output values are provided in Appendix Text S1D. The two approaches applied on WT roots, Approach 1 and 2 (see Methods) were performed on the simulated data (panels A and B, respectively). **A,B** Average value for each simulated root (input) and the value extracted (output) computed using Approach 2 (A), or using Approach 1 (B)) for the root growth rate R_{prod}, the elongation rate r_{elong} and the time cells spend on the EZ t_{resid}. ***(continues in next page)***

continuation of Appendix Figure S5 caption

(A, left) For each simulated root file, exact average (input) values and estimated inferred (output) values are represented. (A, right) Boxplots of the values shown in left panel. In B, the outputs were all computed through Approach 1 and hence only average values with an error estimation were extracted for the whole set of simulated roots. Two values of output are provided which only differ on how the average of the root growth rate (extracted through Approach 1) was computed: 1. by performing a best linear fit on the root length over time of each simulated root file, extracting the slope of this fit and averaging it for all simulated roots and setting this average slope as the average root growth rate, 2. by averaging the length of all the simulated roots at four distinct time points, performing a best linear fit on these averaged data, and setting the slope of this fit as the average root growth rate. For both methods, production rate R_{prod} was extracted as the ratio between the average root growth rate (R_{root}) and the average length of the mature cells (those in the DZ, $<l_{diff}>$), $R_{prod}=R_{root}/<l_{diff}>$; The elongation rate as $r_{elong}=<\ln(r_{EZ,j})>R_{prod}$ and the residence time in the EZ $t_{resid}=<N_{EZ,j}>/R_{prod}$ (corresponding to the formulae in Table EV2 of Approach1. Brackets stand for averages over all root data). Simulations were done using the values of parameters described in Table EV3 (Generic Case) except for the threshold of differentiation, which was considered to be variable from cell to cell within the same root file, in order to asses the method with additional variability. The threshold cell length for any cell in any root file was computed from a Gaussian distribution with mean 175.5 µm and standard deviation 39 µm. n=121 roots were simulated. Boxplots represented as in Appendix Fig. S1.

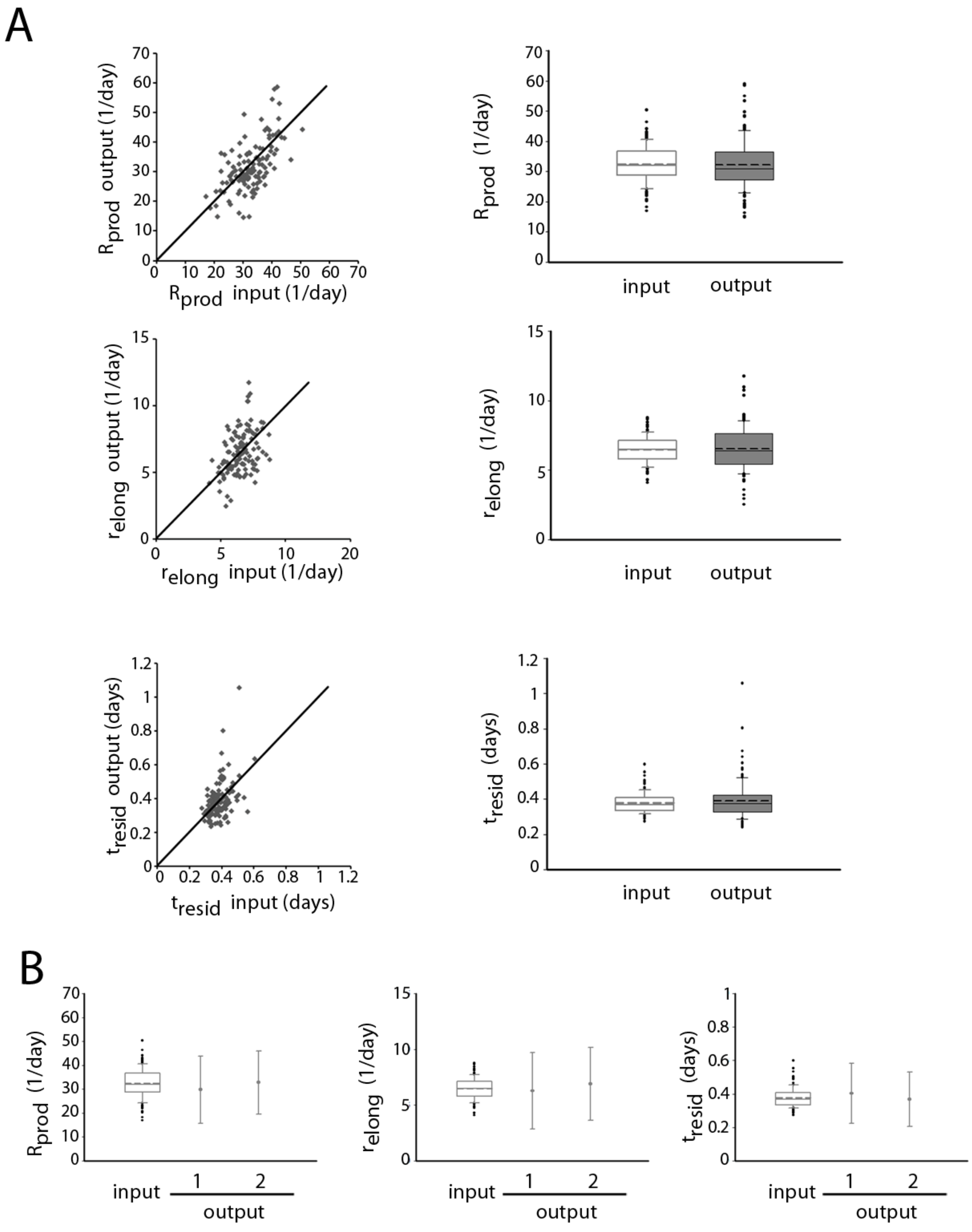

Appendix Figure S6. Validation of the methods used to infer dynamical information, using the computational data of the Ruler model.

The same type of data as those shown in Appendix Fig. S5 but for the Ruler model. **A,B** Average value for each simulated root (input) and the value extracted (output) computed using Approach 2 (A), or using Approach 1 (B)) for the root growth rate R_{prod}, the elongation rate r_{elong} and the time cells spend on the EZ t_{resid}. (A, left) For each simulated root file, exact average (input) values and estimated inferred (output) values are represented. (A, right) Boxplots of the values shown in left panel. In B, the outputs were all computed through Approach 1 and hence only average values with an error estimation were extracted for the whole set of simulated roots. Two values of output are provided which only differ on how the average of the root growth rate (extracted through Approach 1) was computed: ***(continues in next page)***

continuation of Appendix Figure S6 caption

1. by performing a best linear fit on the root length over time of each simulated root file, extracting the slope of this fit and averaging it for all simulated roots and setting this average slope as the average root growth rate, 2. by averaging the length of all the simulated roots at four distinct time points, performing a best linear fit on these averaged data, and setting the slope of this fit as the average root growth rate. For both methods, production rate R_{prod} was extracted as the ratio between the average root growth rate (R_{root}) and the average length of the mature cells (those in the DZ, $<l_{diff}>$), $R_{prod}=R_{root}/<l_{diff}>$; The elongation rate as $r_{elong}=<\ln(r_{EZ,j})>R_{prod}$ and the residence time in the EZ $t_{resid}=<N_{EZ,j}>/R_{prod}$ (corresponding to the formulae in Table EV2 of Approach1. Brackets stand for averages over all root data). Simulations were done using the values of parameters described in Table EV3 (Generic Case) but with no variability in the differentiation threshold (i.e. $\Delta=0$ for the EZ length) (n=121). Boxplots represented as in Appendix Fig. S1.

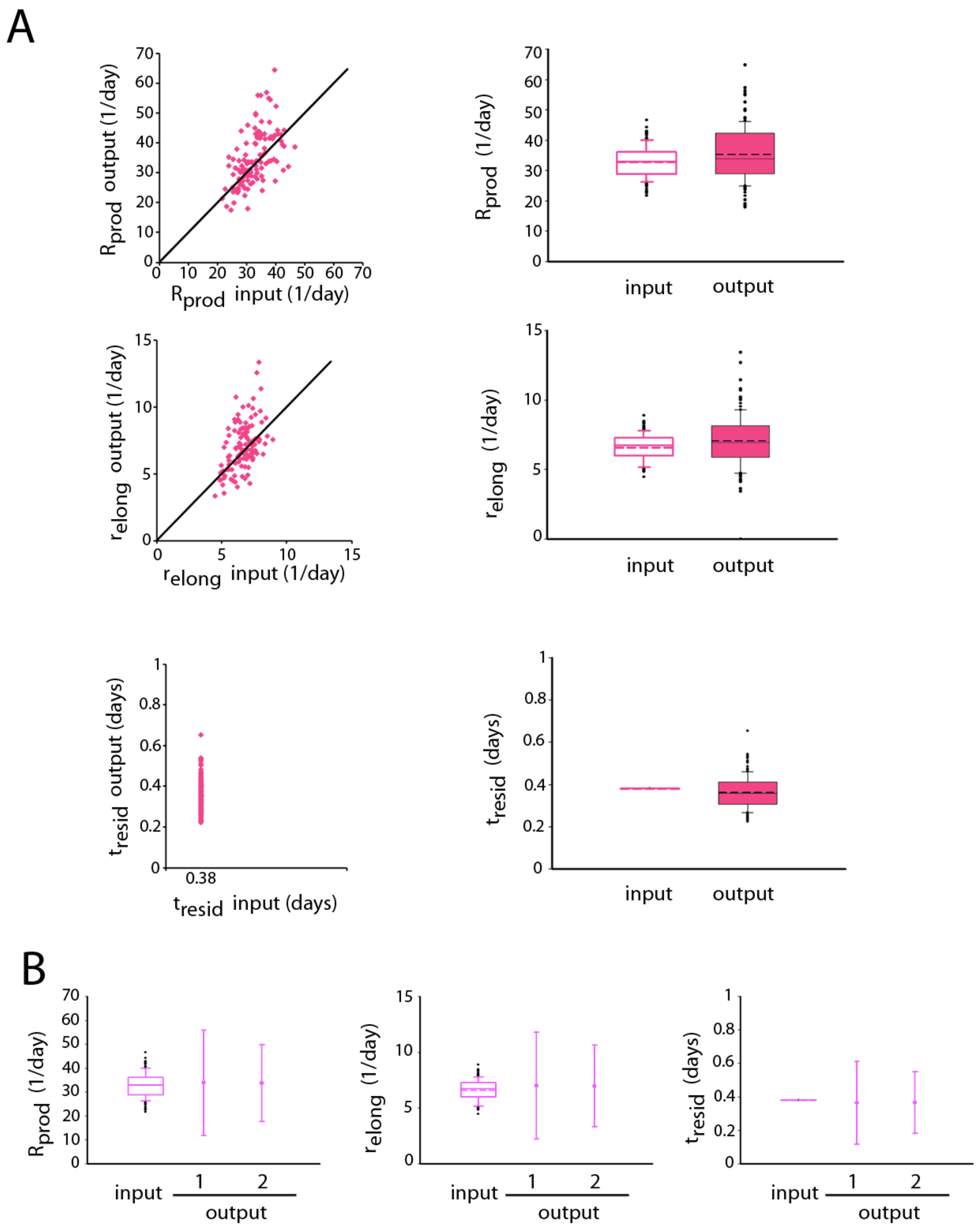

Appendix Figure S7. Validation of the methods used to infer dynamical information, using the computational data of the Timer model.

The same type of data as those shown in Appendix Fig. S5 but for the Timer model. **A,B** Average value for each simulated root (input) and the value extracted (output) computed using Approach 2 (A), or using Approach 1 (B)) for the root growth rate R_{prod}, the elongation rate r_{elong} and the time cells spend on the EZ t_{resid}. (A, left) For each simulated root file, exact average (input) values and estimated inferred (output) values are represented. (A, right) Boxplots of the values shown in left panel. In B, the outputs were all computed through Approach 1 and hence only average values with an error estimation were extracted for the whole set of simulated roots. Two values of output are provided which only differ on how the average of the root growth rate (extracted through Approach 1) was computed: ***(continues in next page)***

continuation of Appendix Figure S7 caption

1. by performing a best linear fit on the root length over time of each simulated root file, extracting the slope of this fit and averaging it for all simulated roots and setting this average slope as the average root growth rate, 2. by averaging the length of all the simulated roots at four distinct time points, performing a best linear fit on these averaged data, and setting the slope of this fit as the average root growth rate. For both methods, production rate R_{prod} was extracted as the ratio between the average root growth rate (R_{root}) and the average length of the mature cells (those in the DZ, $\langle l_{diff} \rangle$), $R_{prod}=R_{root}/\langle l_{diff} \rangle$; The elongation rate as $r_{elong}=\langle \ln(r_{EZ,j}) \rangle R_{prod}$ and the residence time in the EZ $t_{resid}=\langle N_{EZ,j} \rangle / R_{prod}$ (corresponding to the formulae in Table EV2 of Approach1. Brackets stand for averages over all root data). Simulations were done using the values of parameters described in Table EV3 (Generic Case) but with no variability in the differentiation threshold (i.e. $\Delta=0$ for the time cells spend in the EZ) (n=121). Boxplots represented as in Appendix Fig. S1.

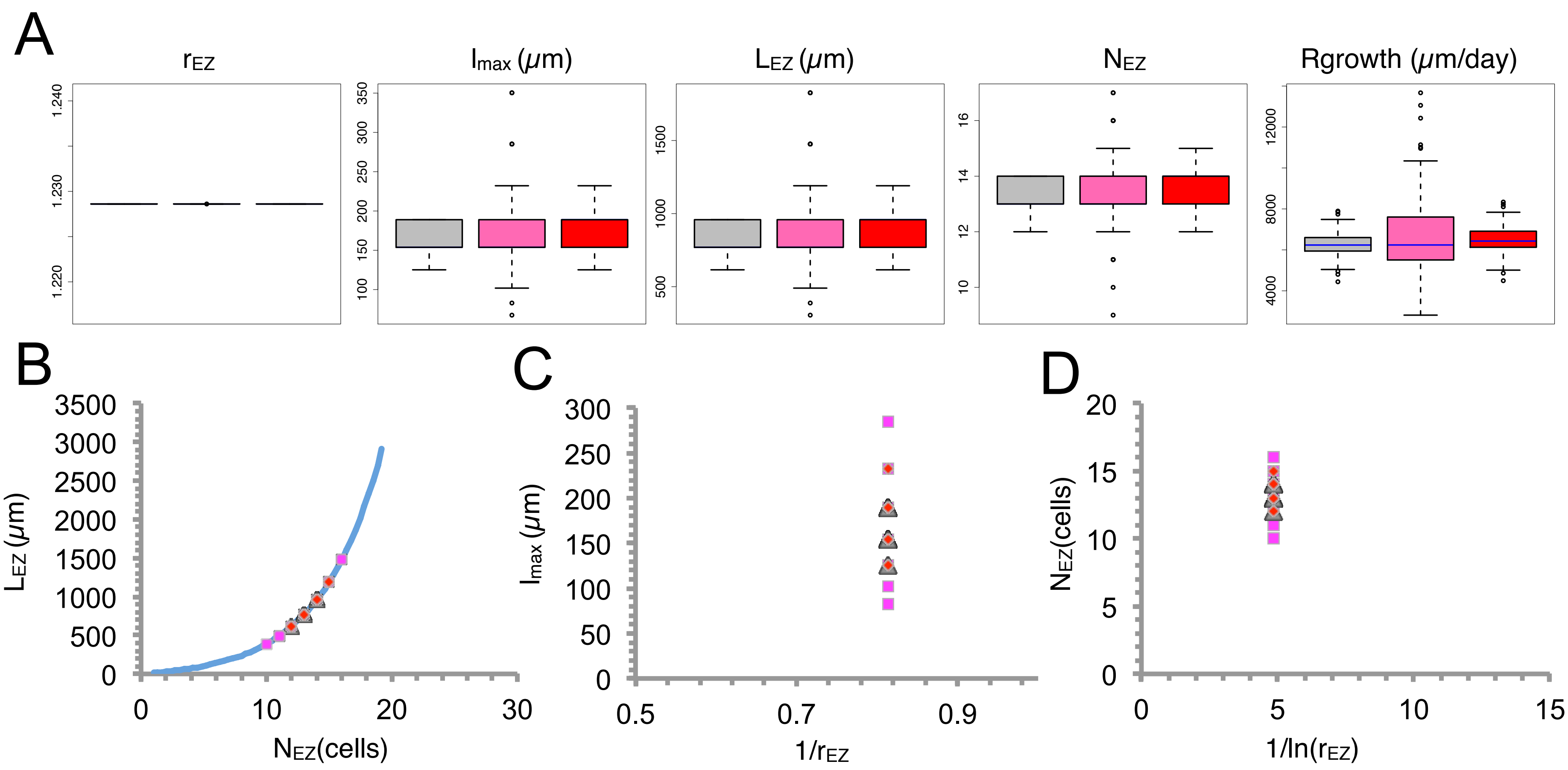

Appendix Figure S8. The three models drive equivalent relationships between phenotypic traits when only the threshold for cell elongation cessation varies between simulated roots (n=121). The simulated roots differ only in the threshold value (of distance for Ruler, of time for Timer and of length for Sizer) that sets cell elongation termination (Methods, Tables EV3). In all models, the threshold value in each root is set from a Gaussian distribution with a standard deviation that is 10% of the mean distribution. Thus all models have the same relative threshold variability of 10%. Simulation results are depicted in gray for Ruler, pink for Timer and red for Sizer models. **A.** Boxplots for phenotypic traits: elongation factor r_{EZ}, length of the EZ cell closest to the DZ l_{max}, length of the elongation zone L_{EZ}, number of cells in the elongation zone N_{EZ} and root growth rate. The Timer model is more sensitive to the threshold variability, driving less reliable root growth rates. No statistical comparison between data is done. **B-D** Relationships between pairs of phenotypic traits for the Ruler (gray triangles), Timer (pink squares) and Sizer (red diamonds) models. Symbols represent simulated data. Continuous blue line is the theoretical prediction which is the same for all three models (Appendix Text S1E). Parameter values are those in Generic Case-Table EV3 but with null variability between cells and roots, except for the threshold for cell elongation cessation, with mean L_0=830 µm for Ruler, l_{diff}=190 µm for the Sizer and T_0=0.38 day^{-1} for the Timer, and standard deviation of 10% the mean in each model. Boxplots represented as in Fig. 2.

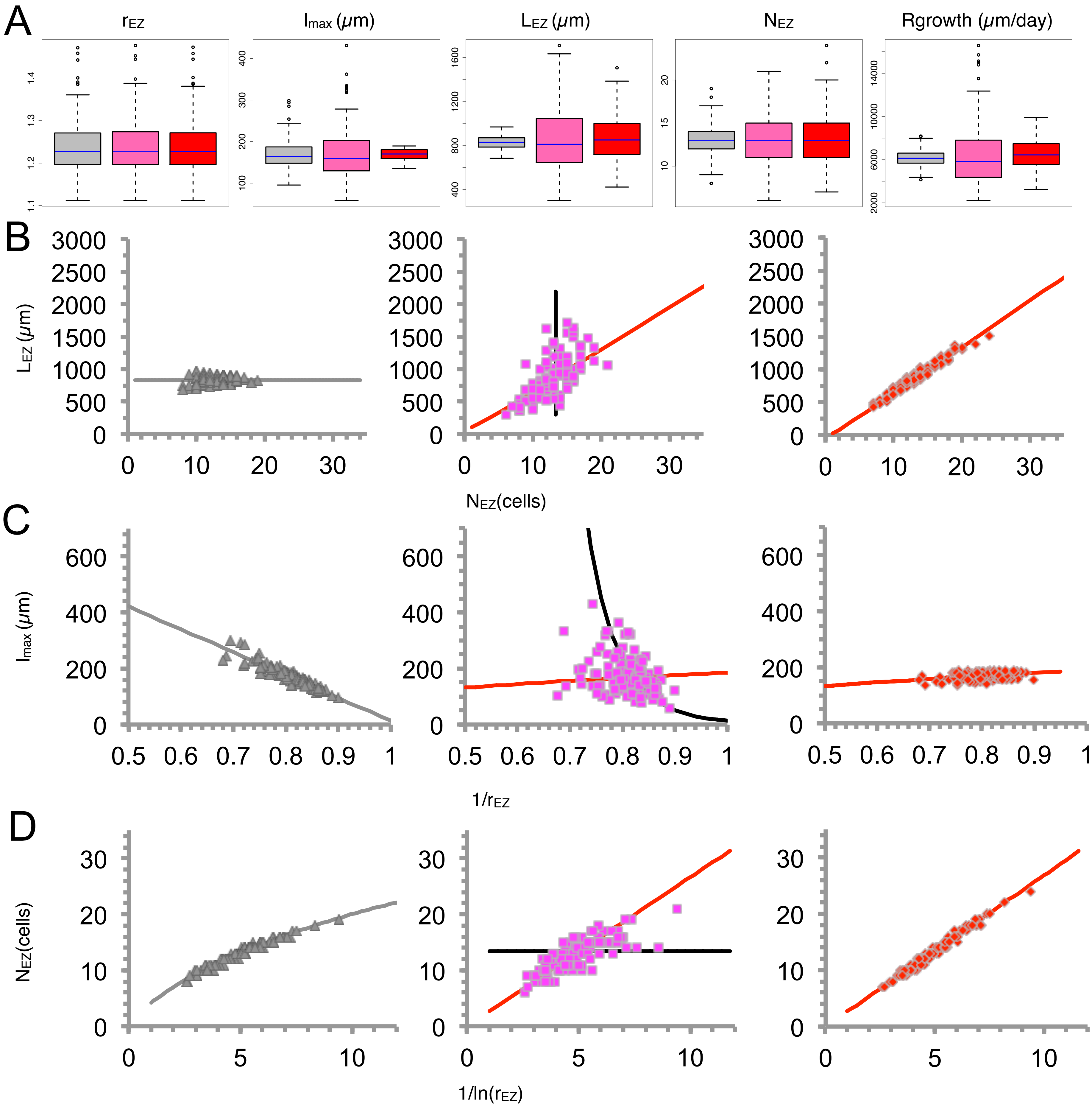

Appendix Figure S9. Model specific relationships between phenotypic traits when the threshold for cell elongation cessation is identical among all simulated roots (n=121). The simulated roots and cells differ only in the cell elongation rates and the meristematic activities, whose values are exactly the same for the three (Ruler, Timer and Sizer) models and are set according to Gaussian distributions (Methods). Simulation results are depicted in gray for Ruler, pink for Timer and red for Sizer models. **A** Boxplots for phenotypic traits: elongation factor r_{EZ} , length of the EZ cell closest to the DZ l_{max}, length of the elongation zone L_{EZ} , number of cells in the elongation zone N_{EZ} and root growth rate. The variability in the elongation rates and meristematic activity is the same in all models and drives the same variability in r_{EZ}. The Timer model is more sensitive to this variability, driving less reliable root growth rates. No statistical comparison between data is done. **B-D** Relationships between pairs of phenotypic traits. Panels from left to right: the Ruler, Timer and Sizer models. Symbols represent simulated data. Continuous lines are theoretical predictions for each model (Appendix Text S1E). In contrast with the Ruler and Sizer models, the theoretical Timer model relationships depend on which factor varies (either the elongation rate (black line) or the meristematic activity (red line)). Parameter values are those Generic Case-Table EV3 except for the variability in the threshold which here is zero. Boxplots represented as in Fig. 2.

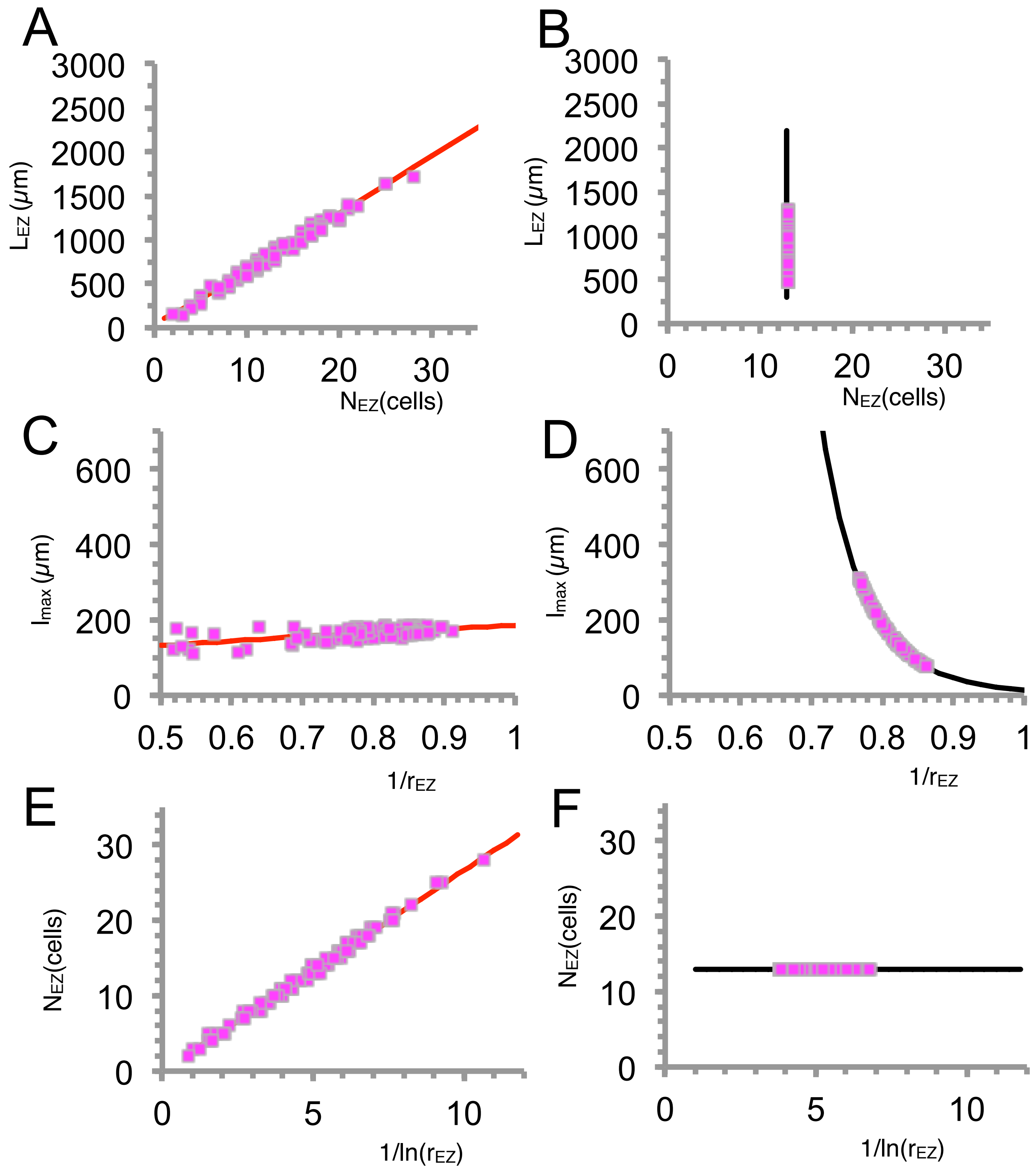

Appendix Figure S10. Dependence of the Timer model on the variability in the elongation rate and the meristematic activity. A,C,E The only parameter that exhibits variability between roots and cells is the meristematic activity. **B,D,F** The only parameter that exhibits variability between roots and cells is the relative cell elongation rate. All results correspond to the Timer model. The symbols represent simulated data. Continuous lines are theoretical predictions (the same lines are depicted in Appendix Fig S9 and in Fig 2 for the Timer model). **A,B** Length of the elongation zone L_{EZ} versus the number of cells in this zone N_{EZ}. **C,D** Length of the EZ cell closest to the DZ l_{max} versus $1/r_{EZ}$. **E,F** Number of cells in the elongation zone N_{EZ} versus $1/\ln(r_{EZ})$. Parameter values like those Generic Case-Table EV3 except for the variabilities in the threshold, the initial cell length and (A,C,E) the cell elongation rate or (B,D,F) which are zero.

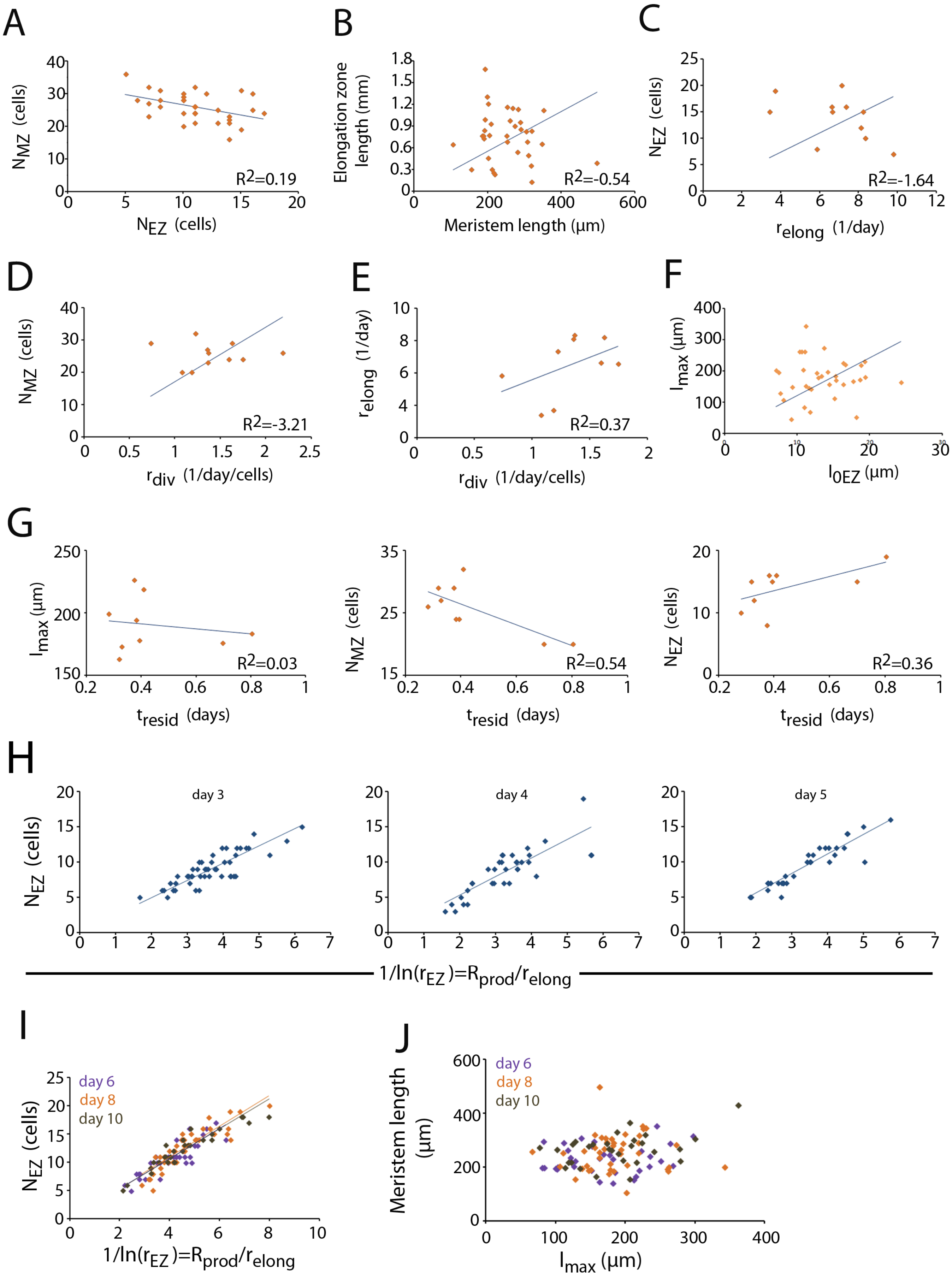

Appendix Figure S11. Relationships between different root phenotypic traits.

A-G. Evaluation of correlations between several growth parameters. For r_{div}, r_{elong} and t_{resid}, data from Approach 2 was used, n=24, 8-day-old WT roots, epidermal file. For all the other parameters, data from Approach 1 was used, n=36, data from 6-day old WT roots, epidermal file (in Dataset EV2). **H-I**. Correlations between the number of epidermal cells in the elongation zone and the ratio between the production rate and the elongation rate (meristem-elongation correlation) before stationary growth (H) and at stationary state (I). Symbols represent data from 30 plants per each day (dpg) being indicated. (J). Meristem length as a function of the mature cell length at stationary state. No correlation could be found in WT between these parameters. Symbols represent data from 30 plants per each day (dpg) being indicated. Data are from Col-0 WT epidermis in Dataset EV2.

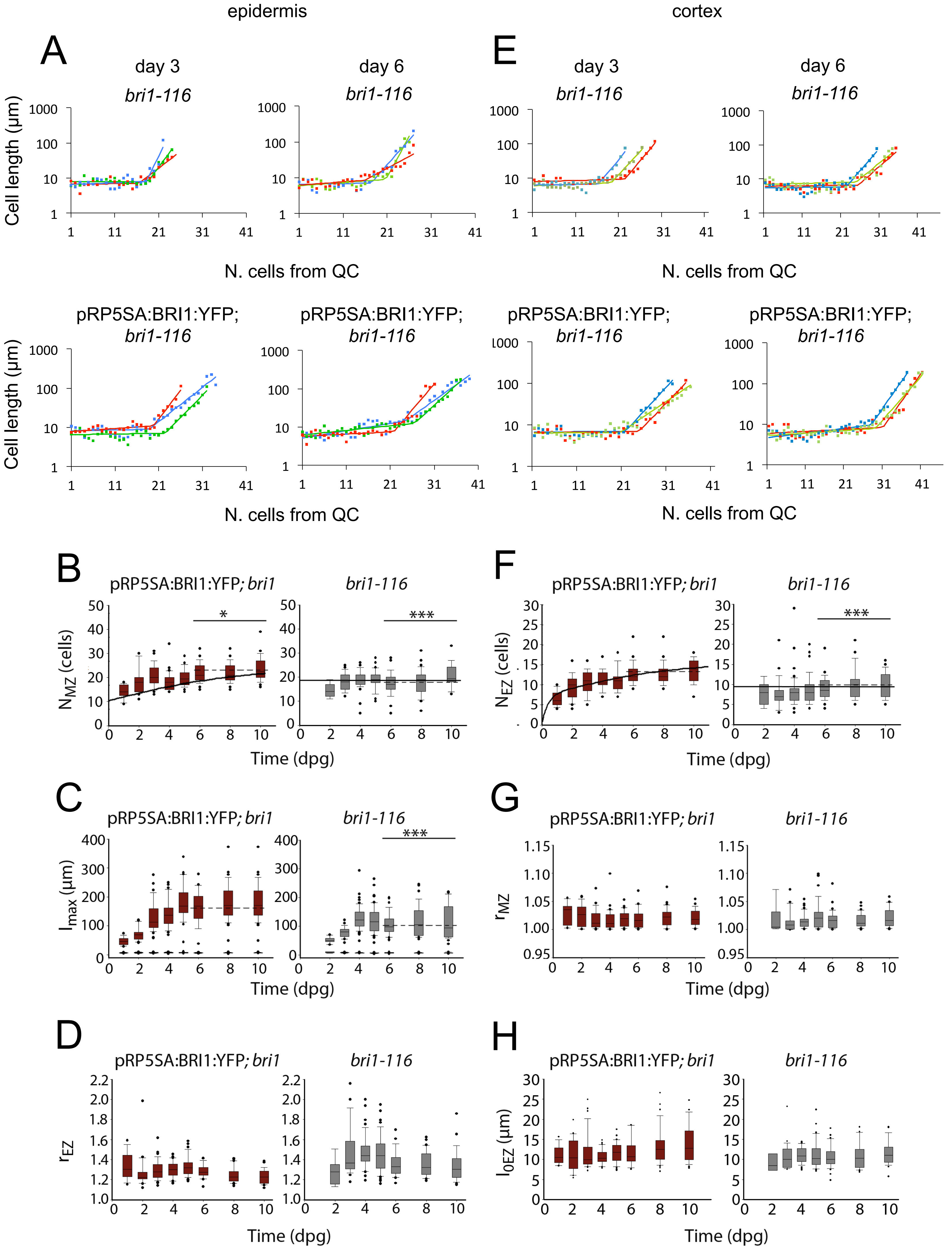

Appendix Figure S12. Quantification of *bri1-116* mutant and pRP5SA:BRI1:YFP;*bri1-116* line.

A, E. Length of epidermal (A) and cortex (E) cells from 3 and 6-day-old *bri1-116* and ***(continues in next page)***

continuation of Appendix Figure S12 caption

pRP5SA:BRI1:YFP;*bri1-116* plants, as a function of the position relative to the QC, for 3 different individual plants (symbols). Straight lines stand for the fitted curves assuming an exponential behavior in each zone and their connection. The slope of each fitted curve is the growth parameters r_{MZ} (for the meristematic zone) and r_{EZ} (for the elongation zone; also called elongation factor). For cell length axis logarithmic scale is used.

B-D, F-H. Data from epidermal cells for *bri1-116* mutant and pRP5SA:BRI1:YFP;*bri1-116* line. Number of cells in the meristem (B) and the elongation zone (F), length of the EZ cell closest to the DZ (C), growth parameters (D,G), and length of the last meristematic cell (H) over time. Black dashed lines represent the average value characterizing the steady state, computed from the values at days 6,8 and 10 day post germination (dpg). Black continuous lines mark the tendency. See Table EV1 for number of plants analyzed. *$p<0.05$. Boxplots represented as in Appendix Fig. S1. Data in A, E are the *bri1-116* epidermal data in Dataset EV1. Data in B-D, F-H are the *bri1-116* epidermal data in Dataset EV2.

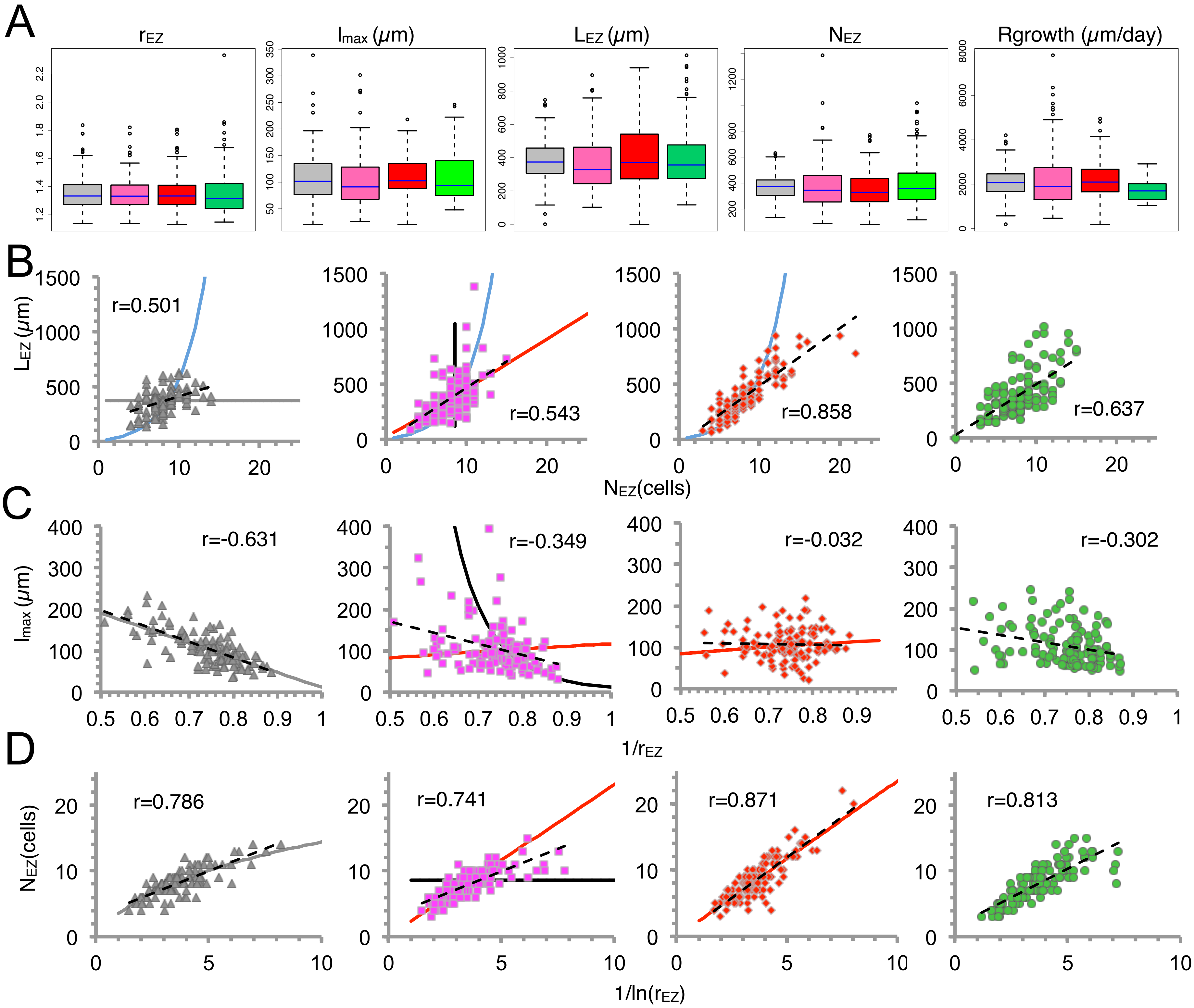

Appendix Figure S13. Comparison between the predictions of three models for final differentiation with empirical data from the epidermis in *bri1-116* roots. Simulation results are depicted in gray for Ruler, pink for Timer and red for Sizer models (n=125, each). Data from epidermis (trichoblast) in *bri1-116* roots is in green (n=126, day 6,8, and 10 post germination (Approach 1), and day 8 post germination (Approach 2) in Dataset EV2, summarized in Table EV5). **A** Boxplots for phenotypic traits: elongation factor r_{EZ} , length of the EZ cell closest to the DZ l_{max}, length of the elongation zone L_{EZ} , number of cells in the elongation zone N_{EZ} and root growth rate. For the epidermis *bri1-116* data, the first four phenotypic traits are all measured in the same root files (n=126), while the root growth rate is measured on a different set of roots (n=12, Table EV7). Statistical comparison (Wilcoxon rank-sum test) of each modelled phenotypic trait (for n=1000) with the *bri11-16* trait (n=126) gives p-values >0.01 for all traits (Table EV4). **B-D** Relationships between pairs of phenotypic traits. Panels from left to right: the Ruler, Timer and Sizer models, and empirical epidermis in *bri1-116* roots. Symbols in the three left-most panels represent simulated data. Continuous lines are theoretical predictions for each model. In contrast with the Ruler and Sizer models, the theoretical Timer model relationships depend on which factor varies (either the elongation rate (black line) or the meristematic activity (red line)). Dashed lines are minimum square linear fits. Pearson correlation coefficient r. Parameter values for simulations in A-D detailed in Table EV3. For each model, the simulated roots and cells differ in the threshold value for cell elongation termination, the cell elongation rates, the meristematic activities and the initial cell length. The parameter values are the same for the three models except for the threshold for cell elongation termination, which has relative variability of 32% (Ruler), 2% (Timer) and 33% (Sizer). The epidermis *bri1-116* panels in B-D are exactly the same as those shown in Fig. 5 C, E, G, respectively. Boxplots represented as in Fig. 2.

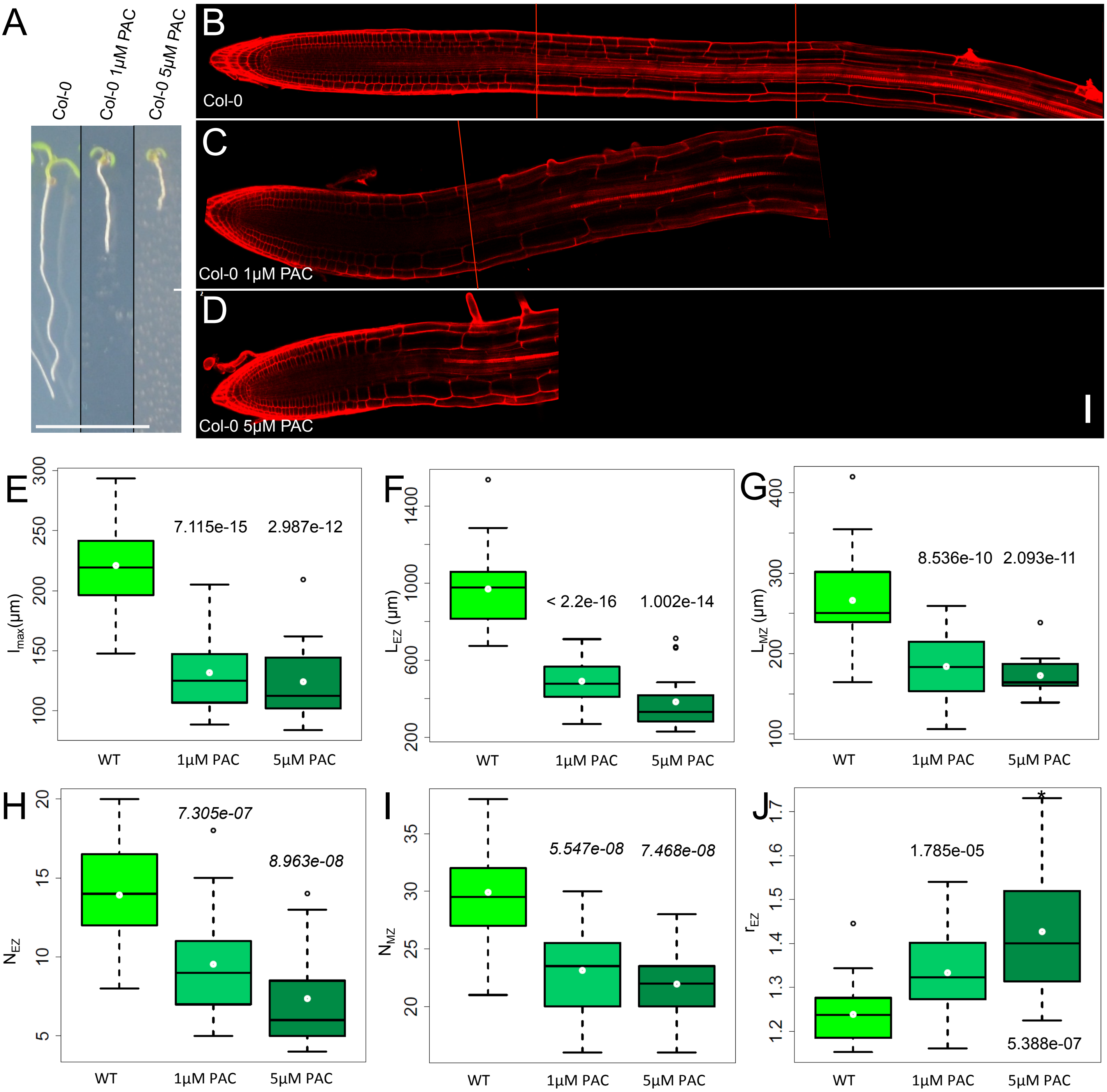

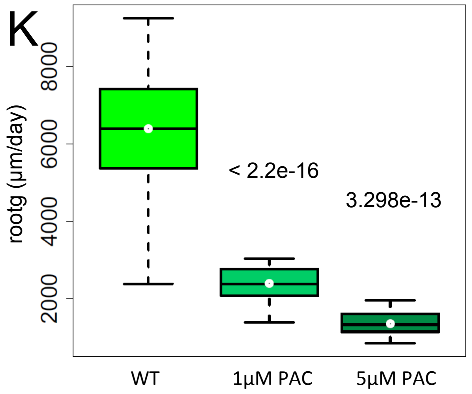

Appendix Figure S14. A 6 day-old-seedlings of Arabidopsis in control conditions (WT), 1µM PAC and 5µM PAC. Scale bar: 1cm **B-D** Confocal images of 6-day old Arabidopsis roots, control conditions, 1µM PAC and 5µM PAC, stained with PI. Scale bar: 50µm. Red bars represent the borders of different images juxtaposed. **E-K** Boxplots of phenotypic traits for control, 1µM PAC and 5µM PAC. The numbers inside each panel denote the p-value of Wilcoxon rank sum test performed on control and 1µM PAC data and on control and 5µM PAC data. For E-J, n=40 for control, n=28 for 1 µM PAC and n=19 for 5µM PAC. **E** Cell length of the EZ cell next to DZ**. F** Length of EZ. **G** Length of meristem. **H** Number of cells in EZ. **I** Number of cells in meristem. **J** Elongation factor in the EZ. **K** Root growth rate. Individual roots were measured each day from day 4 to day 10, both included, post germination. A leat square linear fitting was done for each root. The slope of each fitting gives the root growth rate of each (n=63 control, n=40 for 1µM PAC, n=19 for 5 µM PAC). The data in E-K are those of PAC treatment in Dataset EV2, which are also used in Figure 6 and Appendix Figures S15 and S16.

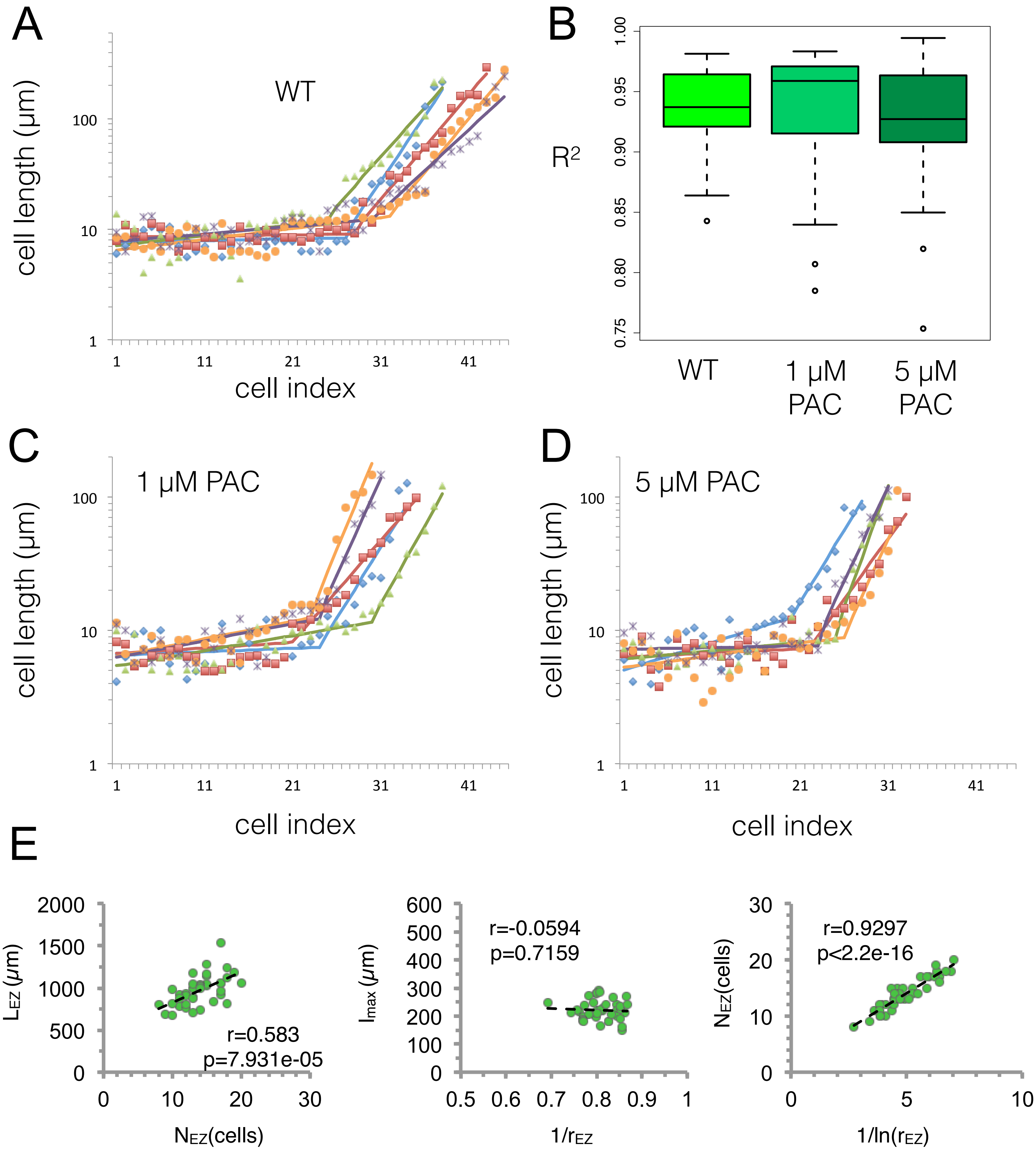

Appendix Figure S15. Fittings to extract the MZ and EZ in the cortex of WT roots and roots treated with PAC.

A,C,D. Length of cortical cells from 6-day-old (A) WT control roots, (C) WT roots grown in 1 µM PAC and (D) WT roots grown in 5 µM PAC as a function of the position relative to the QC, for 5 different individual plants (colored symbols). Logarithmic scale for cell length axis is used. Straight lines stand for the fitted curves assuming an exponential behavior in a linear scale in each zone (as described in Appendix S1B ad using program code in Appendix S3A) and their connection. **B.** Coefficient of determination of the linear fitting done in the EZ. Boxplots represented as in Fig. 2. **E.** Relationships between pairs of phenotypic traits for cortex data of the 6-day-old Col-0 WT roots (n=40) used as control for the experiments with PAC treatment (see Methods). Data in A,C, D are from the PAC treatment data in Dataset EV1. Data in B, E are from the PAC treatment data in Dataset EV2.

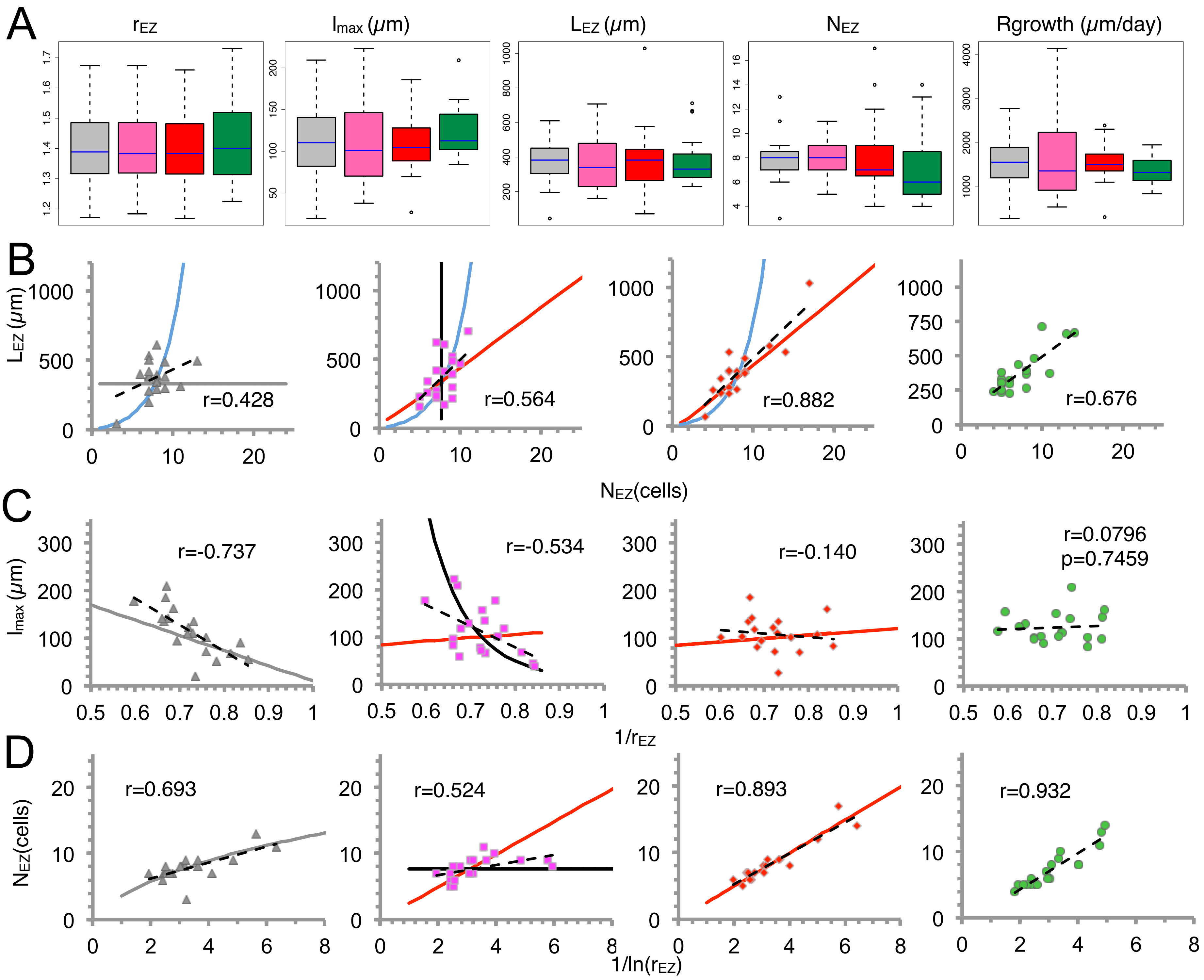

Appendix Figure S16. Comparison between the predictions of three models for final differentiation with empirical data from the cortex tissue in Arabidopsis thaliana roots grown with 5µM PAC. Simulation results are depicted in gray for Ruler, pink for Timer and red for Sizer models (n=19, each). Data from cortex tissue in Col-0+5µM PAC is in green (n=19). **A** Boxplots for phenotypic traits: elongation factor r_{EZ} , length of the EZ cell closest to the DZ l_{max}, length of the elongation zone L_{EZ} , number of cells in the elongation zone N_{EZ} and root growth rate. Timer model is more sensitive to the variability, driving less reliable root growth rates. Statistical comparison (Wilcoxon rank-sum test) of each modelled phenotypic trait with the *bri1l-16* trait gives p-values >0.01 for all traits and models (p-values in Table EV4). **B-D** Relationships between pairs of phenotypic traits. Panels from left to right: the Ruler, Timer and Sizer models, and empirical cortex Col-0+5µM PAC data. Symbols in the three left-most panels represent simulated data. Continuous lines are theoretical predictions for each model (see Appendix Text). In contrast with the Ruler and Sizer models, the theoretical Timer model relationships depend on which factor varies (either the elongation rate (black line) or the meristematic activity (red line)). Dashed lines are minimum square linear fits. Pearson correlation coefficient r for each pair of data. p stands for the p-value. For each model, the simulated roots and cells differ in the threshold value for cell elongation termination, the cell elongation rates, the meristematic activities and the initial cell length (Table EV3). The parameter values are the same for the three models except for the threshold for cell elongation termination, which has relative variability of 27% (Ruler), 1.5% (Timer) and 25% (Sizer) (Table EV3). Boxplots represented as in Fig. 2.

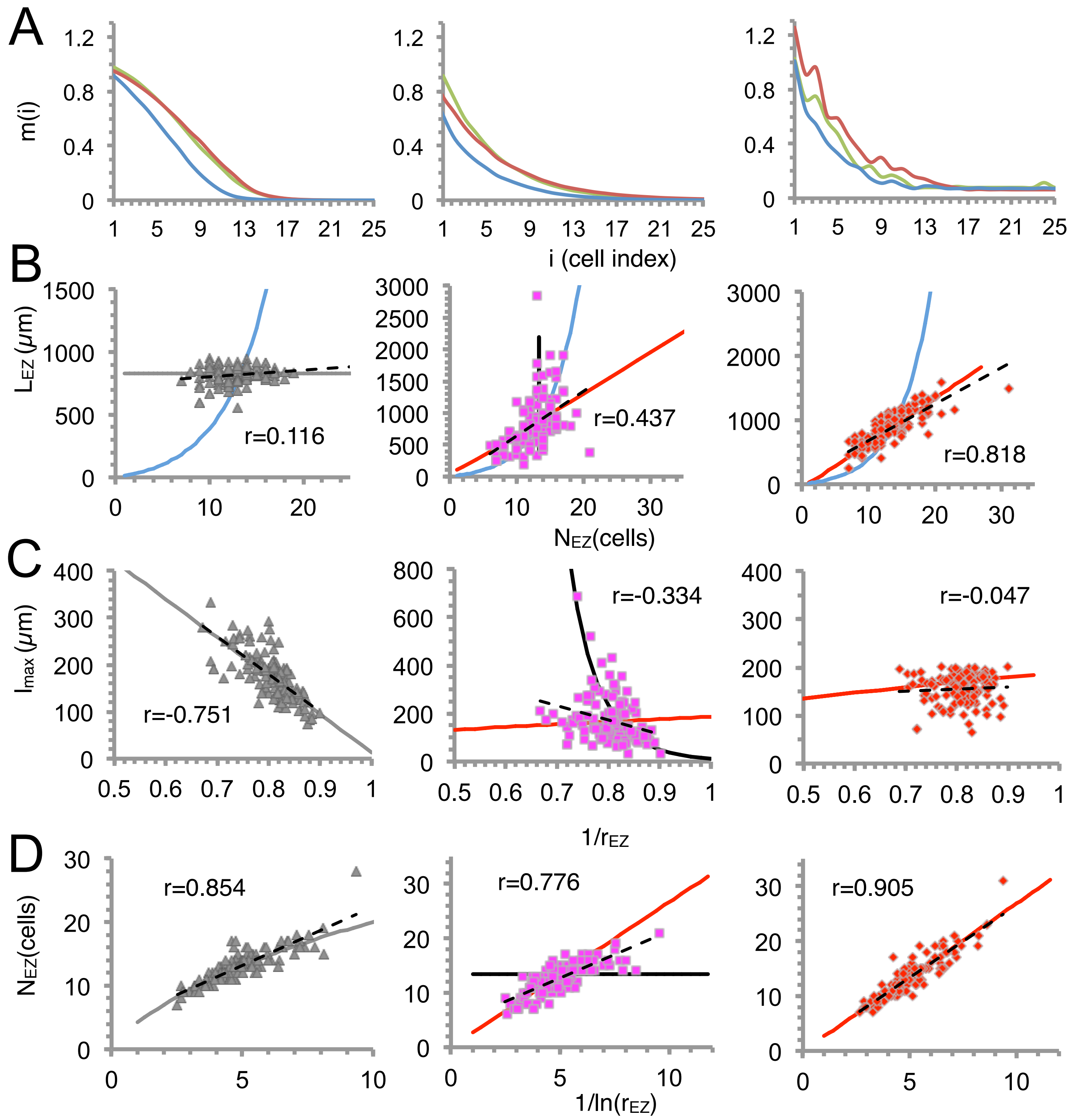

Appendix Figure S17. Ruler, Timer and Sizer mechanisms mediated by signaling gradients. From left to right: results from three models which differ in the type of signaling gradient. The data in Right B-D panels show the type of relationships found in the WT (Figs. 2, 3), corresponding to the Sizer mechanism. **A.** Concentration m (in arbitrary units) of the signaling molecule in cell i as a function of the cell ordinal index i along the EZ, for three different simulated root files (denoted in different colors). i=1 is the cell closest to the MZ, i=2 is its adjacent cell further away from the MZ, and so on an so forth (see labeling in Fig. 2A). For all models the cessation of elongation is dictated when the concentration m of signaling molecule is below a threshold value m_0. The dynamics of cell expansion and appearance into the EZ are the same as the ones described in Results section "A model for cell elongation dynamics during stationary root growth", being the same for all three models. The models differ only on which gradient m(i) is set. Left: m(i)=exp(-Ax(i)) with A=0.0033 μm^{-1} and x(i) being the distance of this cell i from the MZ. This is representative of gradients in which diffusion plays a relevant role and hence depend on the distance from the source that creates the signaling molecule. Middle: m(i)=exp(-Bt_i) with B=7.10 $days^{-1}$ and where t_i is the time (days) this cell has been in the EZ. Therefore, this model assumes that the signaling molecule is degraded over time, it is not significantly transported, and it remains confined in a compartment that does not change of size. Right: m(i)=C/l(i), with C=13μm where l(i) is the length (μm) of the elongating cell. Thus this model assumes that the signaling molecule is not degraded nor significantly transported, such that its concentration decreases only because the cell elongates. All models assume that the signaling molecule is produced only at the MZ. **B-D** Relationships between pairs of phenotypic traits. Pearson correlation coefficient r for each pair of data is depicted. Panels from left to right ordered according to the model used as in A. The results show that each type of signaling gradient mediates a different mechanism, being the Ruler, Timer and Sizer respectively from left to right. Variability in the threshold between roots is modeled by setting a random m_0 value in each simulated root from a Gaussian distribution of mean 0.065 and variance 0.001. Cell parameter values (not the Threshold) as in Generic Case-Table EV3.

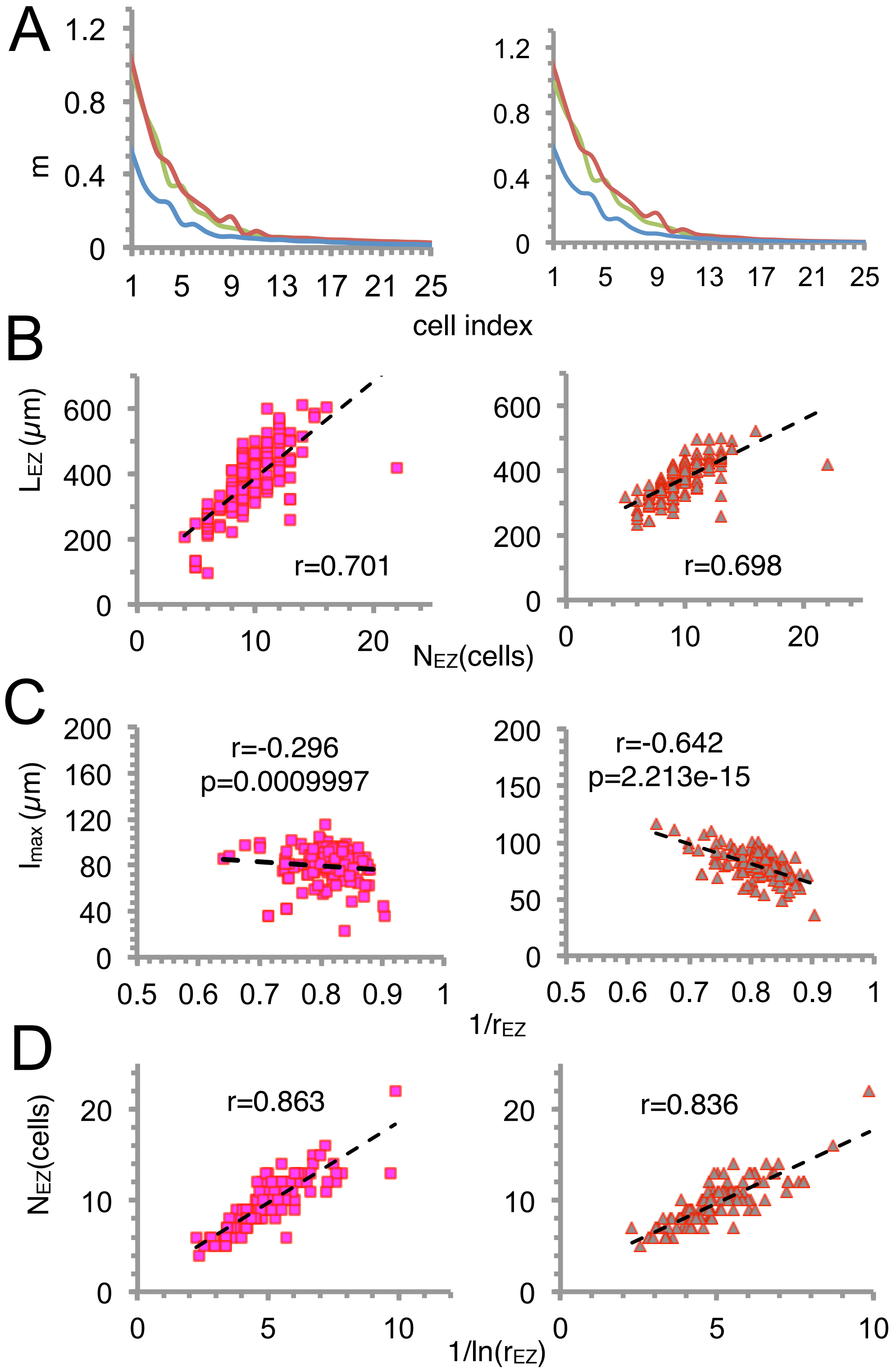

Appendix Figure S18. Mixed signaling gradients that involve dilution because of cell expansion. Left and right panels correspond to two different models. The data in Left panels show the type of relationships found in *bri1-116* mutant (Fig. 5 and Fig. S13). **A.** Concentration m (in arbitrary units) of the signaling molecule as a function of the cell ordinal index along the EZ, for three different simulated root files (denoted in different colors) as in Fig. S17. For the two models the cessation of elongation is dictated when the concentration m of signaling molecule is below a threshold value m_0. The dynamics of cell expansion and appearance into the EZ are the same as the ones described in Results section "A model for cell elongation dynamics during stationary root growth", being the same for the two models. The models differ only on which gradient m(i) is set. Left: This model assumes that the molecule degrades over time, is diluted because of cell expansion and is not significantly transported such that the concentration of the signaling molecule in cell i is $m(i)=C\exp(-Bt_i)/l(i)$ with C=13μm, B=2.5 days^{-1}and l(i) is the length (μm) of the elongating cell. A mixed response between the Timer and Sizer mechanism is found, with less variability than the Timer mechanism. Right: $m(i)=C\exp(-Ax(i))/l(i)$ with with C=13μm, A=0.002 μm^{-1}and l(i) is the length (μm) of the elongating cell. This model is a mix of gradients in Appendix Fig. S17A-Right and Appendix Fig. S17A-Left, which drive a Ruler and a Sizer mechanism respectively. **B-D** Relationships between pairs of phenotypic traits. Left and Right ordered as in A. Pearson correlation coefficient r for each pair of data. p stands for the p-value. Variability in the threshold between roots is modeled by setting a random m_0 value in each simulated root from a Gaussian distribution of mean 0.065 and variance 0.001. Cell parameter values are as in Fig.S17.
